# dCas9-based gene editing for cleavage-free genomic knock-in of long sequences

**DOI:** 10.1038/s41556-021-00836-1

**Published:** 2022-02-10

**Authors:** Chengkun Wang, Yuanhao Qu, Jason K. W. Cheng, Nicholas W. Hughes, Qianhe Zhang, Mengdi Wang, Le Cong

**Affiliations:** 1 Department of Pathology, Stanford University School of Medicine, Stanford, CA USA; 2 Department of Genetics, Stanford University School of Medicine, Stanford, CA USA; 3 Center for Statistics and Machine Learning, Department of Electrical and Computer Engineering, Princeton University, Princeton, NJ USA

**Keywords:** Genetic engineering, CRISPR-Cas9 genome editing, DNA damage and repair

## Abstract

Gene editing is a powerful tool for genome and cell engineering. Exemplified by CRISPR–Cas, gene editing could cause DNA damage and trigger DNA repair processes that are often error-prone. Such unwanted mutations and safety concerns can be exacerbated when altering long sequences. Here we couple microbial single-strand annealing proteins (SSAPs) with catalytically inactive dCas9 for gene editing. This cleavage-free gene editor, dCas9–SSAP, promotes the knock-in of long sequences in mammalian cells. The dCas9–SSAP editor has low on-target errors and minimal off-target effects, showing higher accuracy than canonical Cas9 methods. It is effective for inserting kilobase-scale sequences, with an efficiency of up to approximately 20% and robust performance across donor designs and cell types, including human stem cells. We show that dCas9–SSAP is less sensitive to inhibition of DNA repair enzymes than Cas9 references. We further performed truncation and aptamer engineering to minimize its size to fit into a single adeno-associated-virus vector for future application. Together, this tool opens opportunities towards safer long-sequence genome engineering.

## Abstract (web summary)

Wang, Qu et al. developed a genome-editing system, utilizing catalytically inactive Cas9 fused to microbial single-strand annealing proteins, for kilobase-scale insertion in human cells without introducing DNA nicks or breaks.

## Main

Since the initial demonstration of clustered regularly interspaced short palindromic repeats (CRISPR)–CRISPR associated protein 9 (Cas9) genome engineering, gene-editing technologies have gained broad applications in basic and translational settings^1–10^. New generations of tools have substantially improved the efficiency and fidelity of gene editing and are powerful for altering relatively short sequences^11^. Most gene-editing tools work by cleaving genomic DNA to induce single-strand nicks or double-stranded breaks (DSBs) that facilitate targeted editing^12,13^. These DNA modifications can be repaired by error-prone endogenous pathways such as non-homologous end-joining (NHEJ)^12^. This often leads to unwanted mutations and off-target effects, which could result in toxicity and raise safety concerns^14–18^. Although recent advances in using messenger RNA/protein with optimized donors enhanced levels of efficiency^12^, the editing errors and off-target effects would become increasingly severe when engineering long sequences (≥100 base pairs (bp)). These unwanted effects limit the application of gene editing for genomic knock-in or in vivo gene therapy^19–21^.

Available methods for long-sequence editing, such as homology-directed repair (HDR) and microhomology-mediated end-joining (MMEJ), rely on DNA cutting and often trigger random indel formation^12,13,20^. Recent efforts have enhanced long-sequence editing precision, using chemical enhancers, fusion of enhancement domains or modified donors^22–24^. Nicking-based HDR has been shown to reduce errors but could lead to a lower efficiency^13^. Thus, there remains a need for efficient, safer CRISPR editing tools for long-sequence alterations^19,21^.

Bacteriophages have evolved enzymes that perform precise recombination^25–30^. We reasoned that the key enzyme for microbial recombination, the single-strand annealing protein (SSAP), could be useful for cleavage-free gene editing in mammalian cells. Notably, it does not have DNA-cleavage activity^26,27^; thus, it may not trigger the error-prone pathways needed by Cas9 editing. Motivated by this hypothesis and our previous work showing its ability to stimulate genomic recombination, we developed a gene-editing tool using deactivated Cas9 (dCas9, or catalytically inactive Cas9) and microbial SSAPs^31–38^. This dCas9 editor uses the SSAP for knock-in editing with a donor DNA without the need for DNA cleavage. We termed it dCas9–SSAP editor.

Our data show that dCas9–SSAP has comparable efficiencies to Cas9 references, achieving a knock-in efficiency of up to 20%, and is effective across genomic targets and cell lines for kilobase-scale editing. We also demonstrate dCas9–SSAP knock-in of different transgenes using functional assays. More importantly, our data show that dCas9–SSAP generates near zero on- and off-target errors. When inserting a 1 kb sequence, dCas9–SSAP resulted in less than 0.3% editing errors across the cells sampled, whereas Cas9 editors had similar yields but as much as 10–16% incorrectly edited cells. Across loci, dCas9–SSAP demonstrated an editing accuracy of 90–99.6%, in contrast to editing accuracy in the range of 10–38% for the Cas9 editors. Furthermore, we probed the mechanism of dCas9–SSAP editing by inhibiting DNA repair enzymes and cell-cycle blocking. The results of these assays supported our hypothetical model for a dCas9 editor mediated by SSAP activity when dCas9-guide RNA (gRNA) opens genomic DNAs via the R-loop and are consistent with the known biophysical, biochemical properties of dCas9 (refs. ^39–41^).

Finally, we leveraged structural-guided truncation and aptamer engineering to obtain a minimized dSaCas9–mSSAP editor, achieving a reduction in size of more than 50% and retaining similar levels of efficiency. This minimal dCas9 editor would allow convenient delivery using adeno-associated virus (AAV) vector, which is useful for hard-to-transfect cells or in vivo applications^20,21^. Overall, the dCas9–SSAP editor is capable of efficient, accurate knock-in genome editing. With space for further improvement, it is a valuable cleavage-free gene-editing tool for mammalian cells.

## Results

### Use of phage SSAPs for dCas9 knock-in gene editing

Most CRISPR-based editors capable of long-sequence knock-in require single-strand nicks or DSBs, which can trigger the error-prone NHEJ pathways, resulting in variable efficiency and accuracy^11,12^. In contrast, bacteriophages integrate themselves into host bacteria via recombination systems—for example, lambda Red^42,43^. Such precise phage integration^30,44,45^ relies on a homology-directed step: recombination between genomic and donor DNA stimulated by the SSAPs—that is, lambda Bet or its homologue, RecT^26,46,47^. From previous studies^48,49^, we reasoned that phage SSAPs may not rely on DNA cleavage due to its unusual ATP-independent activity, in contrast to the ATP-dependent RAD51 in mammalian cells^50^. The high affinity of SSAPs for single- and double-stranded DNA may allow attachment to donors when multiple SSAPs are recruited to genomic targets via RNA-guided dCas9 (ref. ^49^). It could then promote DNA exchange without cleavage, as DNA strands become transiently accessible during dCas9-mediated DNA unwinding and R-loop formation^39–41^.

**Fig. 1:**
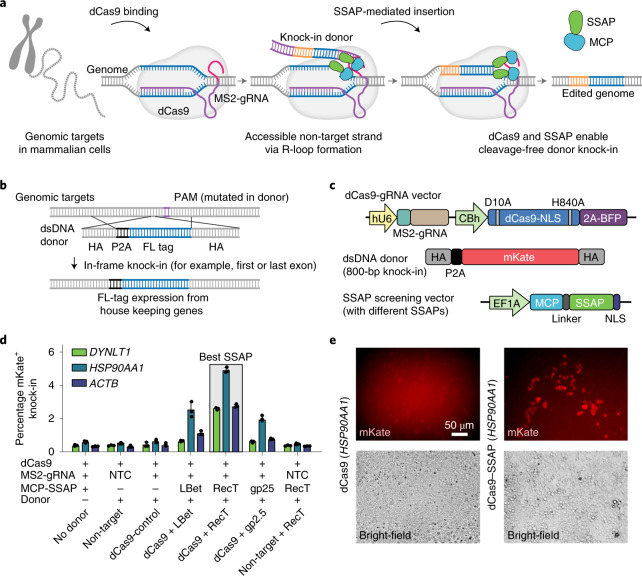
Development of a cleavage-free dCas9-based gene editor using microbial SSAPs. **a**, Schematic model of the dCas9–SSAP editor. **b**, Design of the genomic knock-in assay to measure the level of gene-editing efficiency. FL, fluorescent; PAM, protospacer adjacent motif. **c**, Construct designs for screening the gene-editing efficiency of SSAPs using a genomic knock-in assay with an 800-bp 2A–mKate transgene. NLS, nuclear localization sequence. **d**, Knock-in efficiency of the initial screen of three SSAPs: Bet protein from lambda phage (LBet), RecT protein from Rac prophage (RecT) and gp2.5 from T7 phage (gp2.5). NTC, non-target control. Donor templates with HA lengths of approximately 200 bp (*DYNLT1*) and 300 bp (*HSP90AA1* and *ACTB*) were added in all groups, except the no-donor controls. The error bars represent the s.e.m. of *n* = 3 biologically independent experiments. **e**, Imaging to verify mKate knock-in at endogenous genome loci using the dCas9–SSAP editor. Data represent *n* = 3 biologically independent experiments. dsDNA, double-stranded DNA. Source data

**Extended Data Fig. 1:**
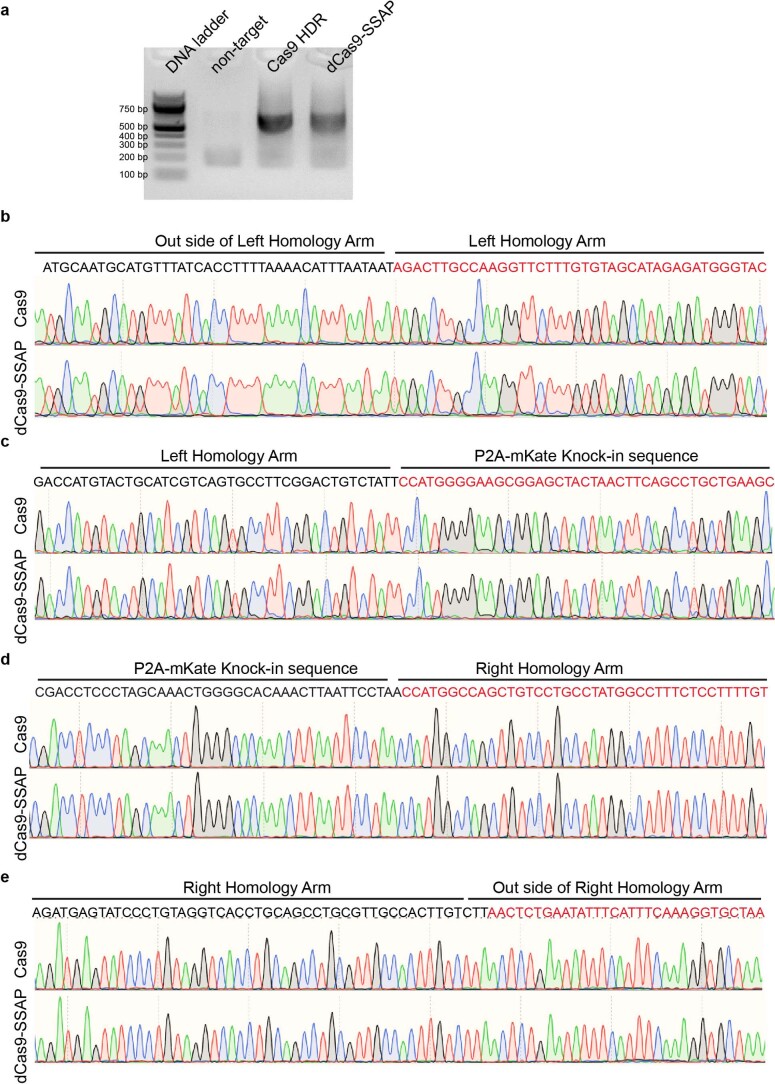
Gel electrophoresis and sequencing verification of knock-in-specific PCR products using dCas9–SSAP. (**a**) Agarose gel results of knock-in-specific junction PCR at *DYNLT1* locus. (**b**–**e**) Sanger sequencing chromatogram of genomic junctions from knock-in experiments at *DYNLT1* locus. For all samples, we amplified the 5′ (**b**, **c**) and 3′ (**d**, **e**) end of genomic DNA using junction-spanning primers outside of the donor DNAs to confirm knock-in. The assay has been performed 3 times with similar results.

Based on this hypothesis, we designed a system to recruit SSAPs to the catalytically inactive dCas9 (Fig. 1a). The dCas9 protein cannot cut DNA but retains the ability to unwind target sites and form an R-loop, rendering the non-target strand putatively accessible for SSAP-stimulated homologous recombination^39,40^. To test this, we engineered and evaluated three major microbial SSAPs: lambda phage Bet, *Escherichia coli* Rac prophage RecT and phage T7 gp2.5 (ref. ^27^). We recruited SSAPs to the deactivated version of *Streptococcus* *pyogenes* Cas9 (dSpCas9, simplified as dCas9 hereafter) via an RNA aptamer MS2 stem-loop (Fig. 1a,c)^31^. This MS2 aptamer was inserted into a single-guide-RNA (sgRNA) scaffold, and the candidate SSAPs were fused to a carboxyl (C)-terminal MS2 coat protein (MCP) that binds specifically to the MS2 aptamer, thus allowing multiple SSAPs to form a complex with dCas9-gRNA. To measure their gene-editing activity in human cells, we generated knock-in donors with an 800-bp transgene encoding a fluorescent protein cassette flanked by homology arms (HAs), which allow in-frame insertion of the fluorescent protein into housekeeping genes, for example, *DYNLT1*, *HSP90AA1* and *ACTB* (Fig. 1b). Following precise knock-in, we measured the percentage of fluorescent protein-expressing cells to quantify the gene-editing efficiency (Fig. 1d). Our test identified that RecT has higher editing activities relative to other SSAPs in human cells, whereas no editing above background was observed with the dCas9-only or non-target controls (Fig. 1d). We validated this knock-in editing using imaging, gel electrophoresis and sequencing (Fig. 1e and Extended Data Fig. 1). This provided evidence that coupling SSAP to dCas9 enables knock-in gene editing.

### Development of dCas9–SSAP as a mammalian gene-editing tool

**Extended Data Fig. 2:**
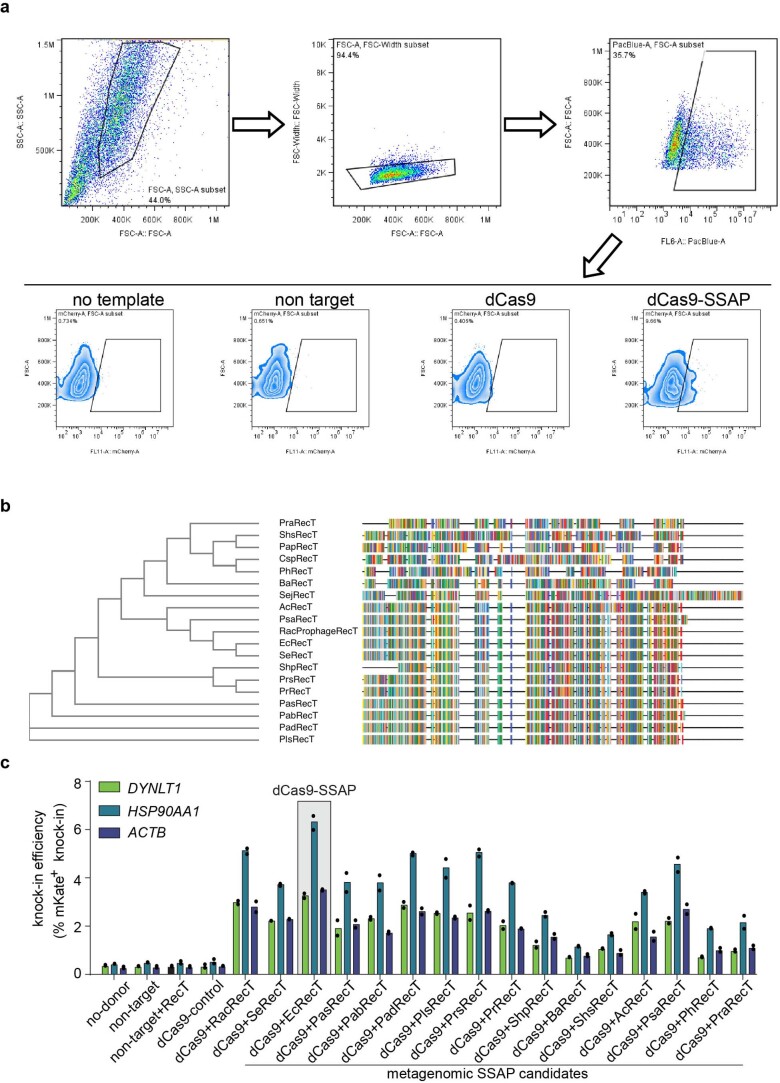
RecT-like SSAP candidate screening. (**a**) Representative FACS plots of gating strategy for the current study. (**b**) Phylogenetic tree and amino acid alignment of representative RecT homologues along with the protein conserved domain annotated. (**c**) Screening RecT like SSAP candidates via metagenomics homologue mining and knock-in assay. The most active candidate is labelled as dCas9–SSAP. Data from 2 biologically independent experiments are shown. Source data

We then conducted metagenomic mining to identify the best SSAP for mammalian gene editing. We focused on RecT homologues and sought to maximize evolutionary diversity via a phylogenetic analysis^27^. We searched the NCBI non-redundant sequence database for RecT homologues and identified 2,071 initial candidates. Next, we built phylogenetic trees, subsampled the evolutionary branches and obtained 16 SSAP candidates (Supplementary Notes and Extended Data Fig. 2). We evaluated the SSAP candidates by measuring the level of editing efficiency across three genomic loci. Among all candidates, EcRecT demonstrated the highest efficiency for dCas9 editing, with an efficiency of approximately 6% in human cells. This was notably higher than the dCas9 controls without SSAP, which were comparable to the no-donor controls, suggesting that dCas9 alone cannot perform efficient knock-in (Extended Data Fig. 2c). We also tested SSAP with a non-target control with gRNA that does not recognize the genomic targets, confirming that expression of SSAP alone is not sufficient for knock-in (Fig. 1d). Together, the proposed dCas9–SSAP editor could enable efficient knock-in in human cells. In what follows, we focus on this top design.

### Characterization of the accuracy of dCas9–SSAP gene editing

The motivation for developing dCas9–SSAP was to perform safer, cleavage-free knock-in editing with the help of SSAP. Thus, we experimentally evaluated the accuracy of dCas9–SSAP for knock-in editing targeting a sequence of approximately 1 kb in length. We measured the on-target errors, off-target insertions, cell-fitness effects and editing yields of dCas9–SSAP in comparison with Cas9 references.

#### On-target errors

There are two types of on-target errors: (1) indel only, where undesired indels are inserted but no template; and (2) imperfect knock-in, where complete or partial template is inserted but indels occur at the knock-in junctions.

**Fig. 2:**
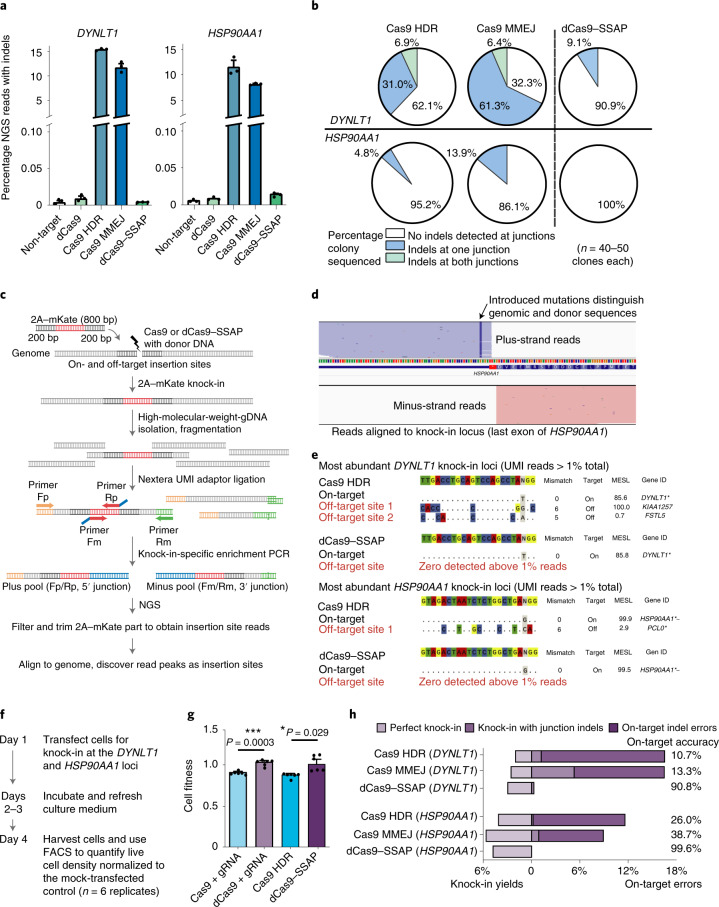
Measurement of the on- and off-target editing errors of dCas9–SSAP. **a**, On-target indel errors (800-kp knock-in). Deep sequencing was used to measure the levels of indel formation when using dCas9–SSAP and Cas9 references at the endogenous targets *DYNLT1* (left) and *HSP90AA1* (right). HDR templates with a 200-bp HA were used as the donor template. Details of the assay are provided in Methods; *n* = 3 biologically independent experiments. **b**, On-target knock-in errors (800 kp knock-in). Clonal Sanger sequencing analysis of the accuracy of knock-in editing using dCas9–SSAP and Cas9 references with different MMEJ and HDR templates. The MMEJ and HDR donor templates had HAs of 25 bp and approximately 200 bp, respectively (Methods). **c**–**e**, Genome-wide detection of insertion sites of the knock-in cassette using unbiased sequencing. The workflow (**c**), representative reads aligned at the knock-in genomic site (**d**) and summary of the detected on-target and off-target insertion sites (**e**) are presented. **f**,**g**, Workflow (**f**) and results (**g**) of the measurement of the cell-fitness effect, defined by the percentage of live cells after editing (normalized to the mock controls). Statistical analyses and comparisons were performed using a Student’s *t*-test; *P < 0.05; ***P < 0.001; *n* = 5 biologically independent experiments. MESL, maximum edit site likelihood. Asterisks next to gene names indicate that the insertion site is within the transcription unit of the gene. **a**,**g**, The error bars represent s.e.m. **h**, Summary of the knock-in accuracy of the dCas9–SSAP editor in comparison with the Cas9 HDR and Cas9 MMEJ methods. Accuracy is defined as the overall yield (%) of correct knock-in in all edited outcomes (correct knock-in, knock-in with indels and NHEJ indels). NGS, next-generation sequencing. Source data

**Extended Data Fig. 3:**
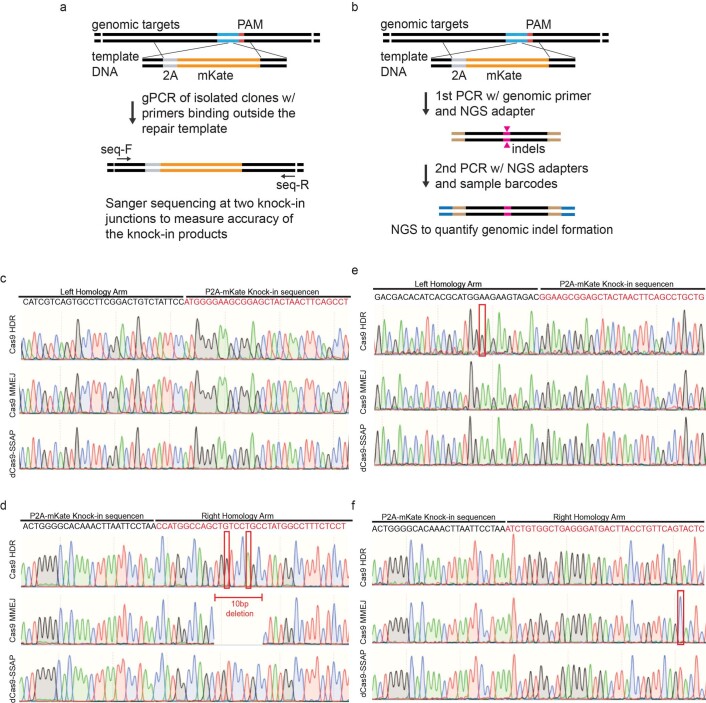
Confirmation of knock-in using sanger sequencing. Schematic showing the workflows used in Sanger sequencing of knock-in products (**a**) and the sequencing method used in deep on-target indel assay (**b**). Assays described here correspond to Fig. 2. gPCR, genomic PCR. Seq-F/seq-R are primers for Sanger sequencing binding upstream/downstream of the knock-in donors. Sanger sequencing chromatogram of genomic junctions from dCas9–SSAP experiments at *DYNLT1* (**c** and **d**) and *HSP90AA1* (**e** and **f**) locus. For all samples, we amplified the 5′ (**c** and **e**) and 3′ (**d** and **f**) end of genomic DNA using junction-spanning primers to confirm knock-in precision. The sequences in the red boxes were not precisely repaired. The genomic-binding primers used are completely outside of the donor DNAs to avoid contamination. The assay has been performed 3 times with similar results.

To evaluate type (1), we used deep sequencing to measure the on-target indel formation of the dCas9 editor. We used a nested PCR design with an initial primer binding site outside the donor DNA to avoid template contamination (Fig. 2a and Extended Data Fig. 3). Deep sequencing showed that the level of on-target errors of the dCas9 editor were as low as those observed for the negative controls, in contrast to high levels of indels observed for the Cas9 editors (Fig. 2a).

To evaluate type (2), we benchmarked the knock-in errors of dCas9–SSAP and measured the junction indels. We clonally isolated the edited cells, amplified the knock-in genomic loci using a similar two-step nested PCR design to avoid contamination (Fig. 2b and Extended Data Fig. 3) and assessed the edited genomic alleles via Sanger sequencing. The long-read Sanger sequencing allowed us to examine the entire knock-in junctions. Our results indicated that, although MMEJ donors were more efficient than HDR donors when using Cas9, they also led to a higher percentage of editing errors (Fig. 2b). More importantly, dCas9–SSAP outperformed Cas9 HDR and Cas9 MMEJ in terms of the percentage of edited clones with no knock-in errors (Fig. 2b and Extended Data Fig. 3). At one locus, dCas9–SSAP achieved 100% knock-in success (within the limit of the assay sensitivity).

#### Off-target errors

**Extended Data Fig. 4:**
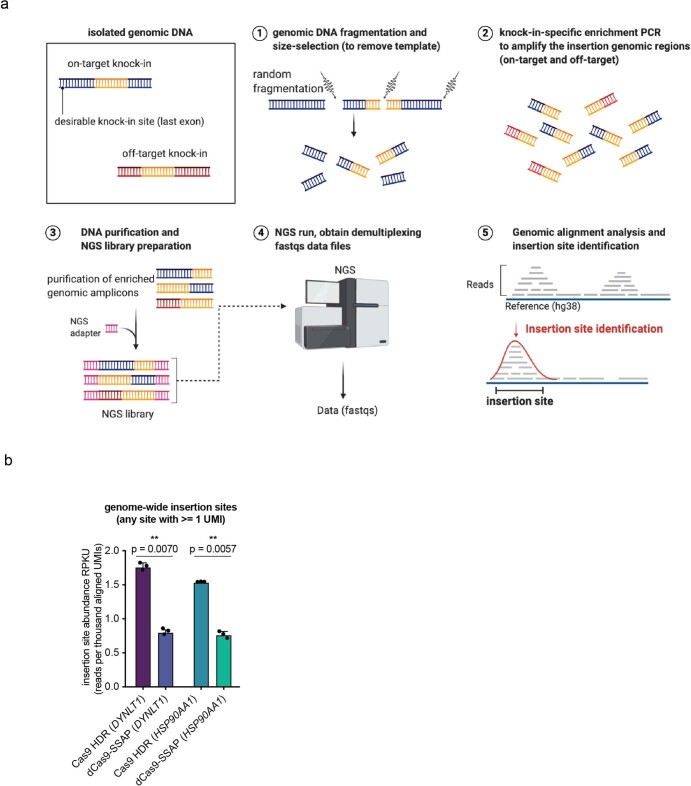
Unbiased genome-wide insertion quantification. (**a**) Overall workflow for unbiased genome-wide insertion site mapping process. On-target and off-target insertions sites are recovered from reads that align to the reference genome (hg38). Full protocol and data analysis pipeline are detailed in Methods. (**b**) Quantification of genome-wide insertion sites counting all aligned reads (with valid UMI) showed decreased insertion site abundance using Cas9-SSAP compared with Cas9 HDR, across two genomic loci (*DYNLT1* and *HSP90AA1*). The abundance of insertion sites is measured as RPKU, or Reads Per Thousand UMIs. n = 3 biologically independent experiments. All results in this figure are from replicate experiments with error bars representing standard error of the mean (S.E.M.). The statistical analysis and comparison were performed using t-test. Source data

We also evaluated the off-target knock-in error rates of dCas9–SSAP editing via a genome-wide transgene insertion assay (Fig. 2c–e and Extended Data Fig. 4a)^31^. Briefly, we isolated high-molecular-weight genomic DNA, followed by fragmentation and unique molecular identifier (UMI)-adaptor ligation, and used transgene-specific primers for the unbiased identification of genomic insertion sites (Fig. 2c). Through a previously validated analysis pipeline modified from a Cas9 genome-wide off-target assay (Methods), we identified enriched peaks of reads that represent high-abundance transgene insertion sites (Fig. 2d). Considering insertion sites with >1% of total aligned reads, our results confirmed that dCas9–SSAP showed no detectable off-target insertions, whereas the Cas9 references led to a substantial number of off-target insertion events (Fig. 2e). Notably, there were fewer off-target sites when we considered all sites with at least one UMI aligned in the dCas9–SSAP samples compared with the Cas9 editor (Extended Data Fig. 4b). This result suggests that dCas9–SSAP could help to address the off-target issues that are prominent in long-sequence knock-in.

#### Cell-fitness effect and editing-yield analysis

We also compared the fitness of cells that went through Cas9/dCas9-based editing. We experimented with two target sites; our data suggested that dCas9 editing leads to higher cell fitness than Cas9 (Fig. 2f,g; defined as the normalized percentage of cells alive after editing).

For the full picture, we summarized the editing yields of dCas9–SSAP in comparison to the Cas9 references. We tabulated the percentages of accurate knock-ins, knock-ins with errors and on-target indels without knock-ins, where the sum of the latter two is the on-target-error total (Fig. 2h). We also measured the overall accuracy of the editing, defined as the ratio of successful knock-in cells to total edited cells (Fig. 2h). We observed that Cas9 editors suffered from frequent errors in long-sequence editing, where the percentage of erroneous edits were notably higher than the yields and the accuracy ranged between 10% and 38%. Although the knock-in yields for dCas9–SSAP were similar to the best Cas9 references, dCas9–SSAP generated minimal errors and achieved an accuracy rate of 90–99% across genomic loci.

### Benchmark dCas9–SSAP across donor designs and cell types

**Fig. 3:**
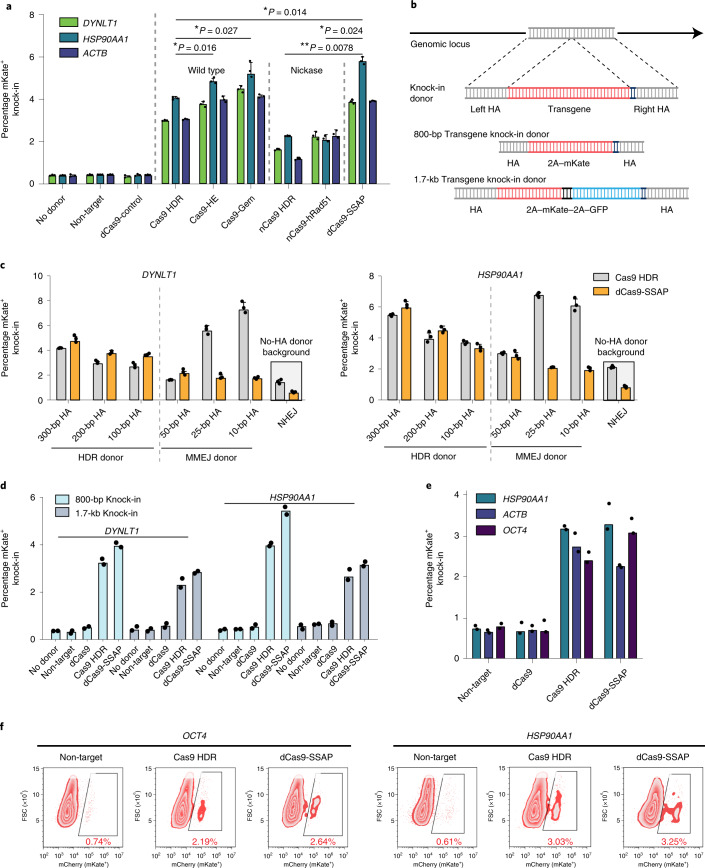
Validation and benchmarking of dCas9–SSAP across donor designs and cell types. **a**, Comparison of the knock-in efficiencies of dCas9–SSAP and other alternative Cas9, nCas9 and HDR-enhancing tools. Cas9-HE, CtIP-fusion Cas9; Cas9-Gem, Geminin-fusion Cas9; nCas9, Cas9-D10A nickase reference; and nCas9-hRAD51, an improved Cas9 nickase editor. The same donor templates as those used in Fig. 1 were used. Statistical analyses and comparisons were performed using a Student’s *t*-test; *P < 0.05 and **P < 0.01. **b**, Design of knock-in donors with different transgene lengths. **c**, Gene-editing efficiencies at the *DYNLT1* (left) and *HSP90AA1* (right) loci in HEK293T cells for three types of donor designs with different HA lengths. **a**,**c**, The error bars represent the s.e.m. of *n* = 3 biologically independent experiments. **d**, Knock-in efficiencies for different transgene lengths using the dCas9–SSAP editors. Donor-HA lengths of approximately 200 bp (*DYNLT1*) and 300 bp (*HSP90AA1*) were used; *n* = 2 biologically independent experiments. **e**,**f**, Knock-in gene editing in hESC (H9) cells using the dCas9–SSAP editor. The knock-in efficiencies of the Cas9, Cas9 HDR and dCas9–SSAP editors (**e**; *n* = 2 biologically independent experiments), and flow cytometry analyses of the Cas9 HDR and dCas9–SSAP editors (**f**) are shown. Donor-HA lengths of approximately 200 bp (*HSP90AA1* and *ACTB*) and 212 bp + 253 bp for *OCT4* were used. Data were collected in duplicate. Source data

**Extended Data Fig. 5:**
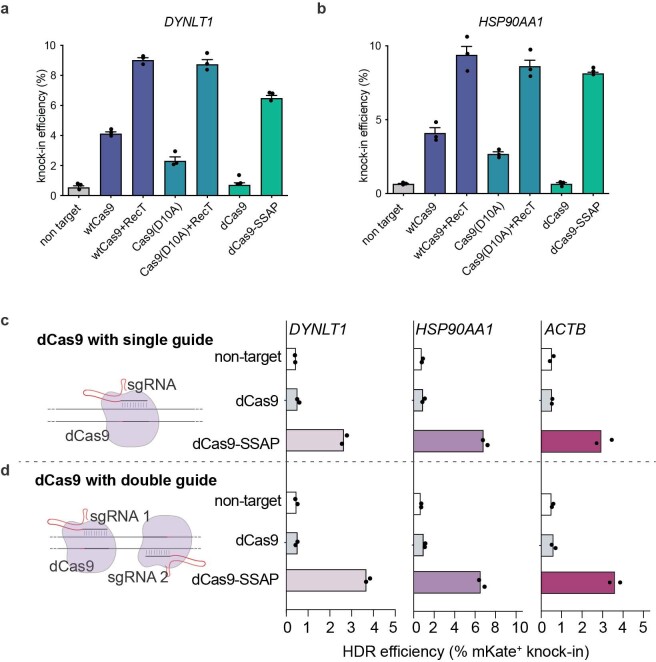
Comparation and optimization of dCas9–SSAP editor. Quantified knock-in efficiency for the comparation of dCas9–SSAP with Cas9/nCas9 based SSAP editor at *DYNLT1* (**a**) and *HSP90AA1* (**b**) locus. The donor DNAs used are the same as shown in Fig. 3a with 800-bp knock-in design. n = 3 biologically independent experiments. All results in this figure are from replicate experiments with error bars representing standard error of the mean (S.E.M.). Testing dCas9–SSAP editor tool using single-guide (**c**) and dual-guide (**d**) designs across three genomic targets (shown on the top). The distance between guides is 19 bp for HSP90AA1, 21 bp for DYNLT1, and 31 bp for ACTB. The donor DNAs used are the same as shown in Fig. 3a with 800-bp knock-in design. Data from 2 biologically independent experiments are shown. Source data

Having established that dCas9–SSAP has higher accuracy in knock-in editing, we further validated its level of efficiency and usages across donor designs and cell types. As benchmarks, we used both wild-type and nicking-based Cas9 (nCas9) editors, including three HDR-enhancing tools^51–53^. We examined their 1-kb knock-in activities across three genomic targets. The comparison demonstrated that dCas9–SSAP achieved higher efficiencies than the Cas9, nCas9 and nCas9-hRAD51 nickase editors, with similar levels of efficiency to Cas9-HE^51^ and Cas9-GEM^52^, two published HDR-enhancing editors (Fig. 3a). We also compared dCas9–SSAP with our previous SSAP-enhanced wild-type Cas9 tools^31^ and found that the dCas9-based editor had robust but reduced activity in comparison to when DNA cleavage was introduced (Extended Data Fig. 5a,b). In addition, our data showed that a single-guide dCas9–SSAP editor was sufficient for effective knock-in, with minor improvements when using two gRNAs (Extended Data Fig. 5c).

**Extended Data Fig. 6:**
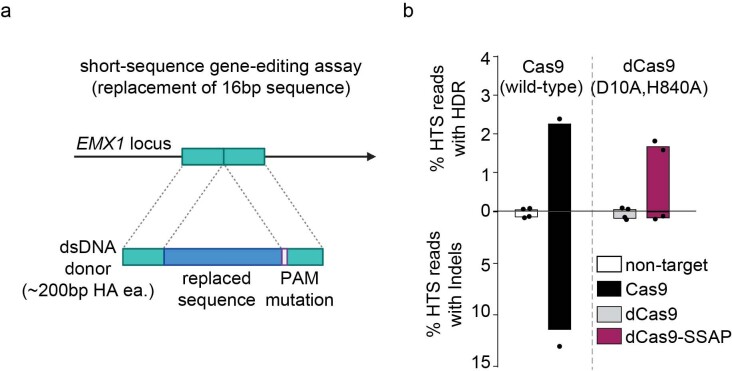
Deep sequencing of short-sequence editing comparing dCas9–SSAP and Cas9 editors. (**a**) donor design of 16-bp replacement at *EMX1*. (**b**) Analyse the precision HDR and indel editing outcomes using deep sequencing at *EMX1* genomic locus. The first round of PCR used sequencing primers completely outside of the donor to ensure the sequencing results will be free from the donor template contamination, validated by the non-target control (where the donor DNAs are delivered into the cells). n = 2 biologically independent experiments.

Next, we tested the dCas9–SSAP editor with different donor DNA designs (Fig. 3b). We first tested the effect of the length of the HA on the efficiency of dCas9–SSAP (Fig. 3c). Our results suggested that SSAP-mediated editing is more efficient when using HDR than MMEJ donors and longer HAs generally result in a higher editing efficiency (Fig. 1f and Supplementary Notes). This is consistent with previous reports that MMEJ relies on DNA breaks, which are missing in dCas9 editing^12,13,54^. We then evaluated the editing efficiency of dCas9–SSAP when the sequence for knock-in has variable length, up to 2 kb for dual-fluorescent protein knock-in (Fig. 3d). Our data showed that dCas9–SSAP performed consistently, with a comparable, and often higher, efficiency to the Cas9 references irrespective of the transgene length (Fig. 3d). In addition, when using a donor that knocked in a 16-bp sequence, we observed dCas9–SSAP supported short-replacement gene editing (Extended Data Fig. 6).

**Extended Data Fig. 7:**
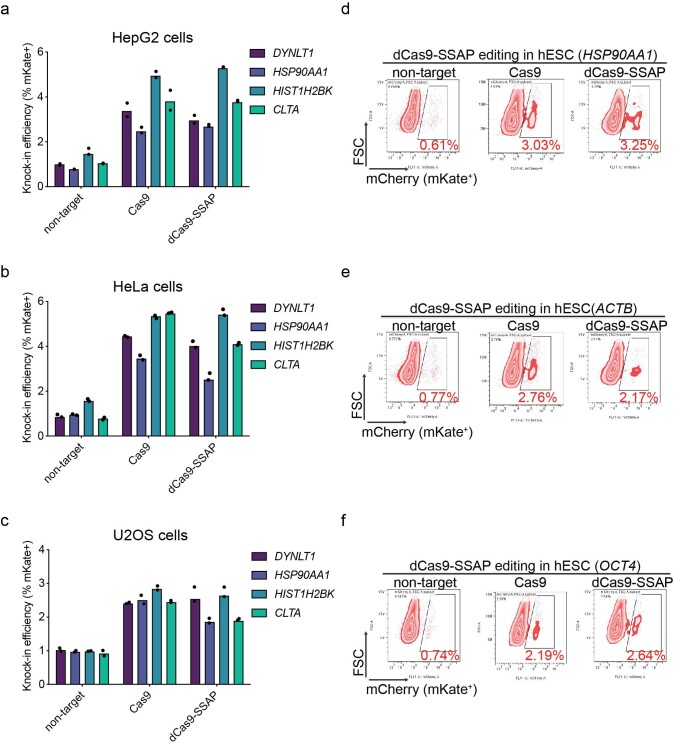
Validation of dCas9–SSAP knock-in efficiencies in different cell lines. (**a**–**c**) Results in HepG2, HeLa, and U2OS cell lines. The knock-in experiments used similar donor DNA with ~800-bp cassettes encoding 2A-mKate transgene for all cell lines tested. n = 2 biologically independent experiments. (**d**–**f**) Flow cytometry analysis of knock-in gene editing at *HSP90AA1*, *ACTB*, *OCT4* endogenous loci in human embryonic stem cells (hESC, H9) using dCas9–SSAP compared with non-target controls and Cas9 (Cas9 HDR) references. Source data

Furthermore, we checked whether the dCas9–SSAP editor can be applied in other cell types beyond the model human embryonic kidney 293T (HEK293T) cell line. We applied dCas9–SSAP to three cell lines with distinctive tissue origins (cervix-derived HeLa cells, liver-derived HepG2 cells and bone-derived U2OS cells). We observed knock-in efficiencies comparable to the Cas9 references in all three lines (Extended Data Fig. 7a–c). Next, we used the dCas9–SSAP editor in human embryonic stem cells (hESCs) to engineer sequences in a more therapeutically relevant setting^19,55^. We observed robust knock-in editing across all three targets (Fig. 3e). To avoid background from donor DNA, the stem-cell editing was performed with short HAs (approximately 200 bp) and an efficiency of about 3% for kilobase-scale editing without selection was achieved. The dCas9–SSAP efficiencies were comparable and often higher than the Cas9 references (Fig. 3f and Extended Data Fig. 7d–f). Thus, we concluded that dCas9–SSAP has similar levels of efficiencies to the Cas9-based editors.

### Optimization of the dCas9–SSAP efficiency for robust knock-in editing

**Fig. 4:**
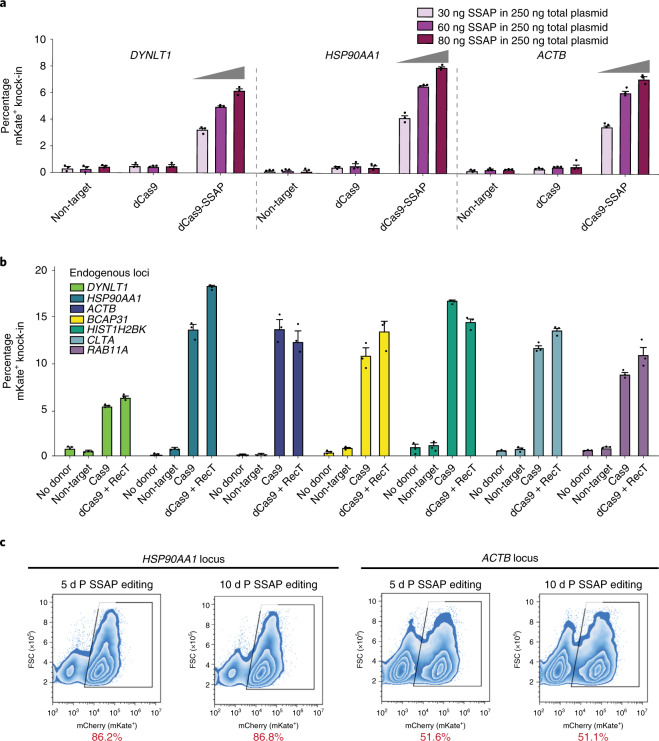
Optimization of dCas9–SSAP for efficient and durable gene editing. **a**, Knock-in efficiencies for SSAP-dosage optimization. Donor-HA lengths of approximately 200 bp (*DYNLT1*), 300 bp (*HSP90AA1*) and 200 bp (*ACTB*) were used; *n* = 3 biologically independent experiments. **b**, Performance (knock-in efficiency) of the dCas9–SSAP editor compared with Cas9 references across seven endogenous loci in HEK293T cells after SSAP-dosage optimization and donor-HA extension. Donor-HA lengths of 673 bp + 750 bp for *HSP90AA1*, 500 bp + 800 bp for *ACTB*, 608 bp + 740 bp for *BCAP31*, 212 bp + 413 bp for *HIST1H2BK*, 705 bp + 602 bp for *CLTA,* 464 bp + 440 bp for *RAB11A* and approximately 200 bp for *DYNLT1* were used. All knock-in donors targeted the carboxy termini of the endogenous proteins, except for the *CLTA* and *RAB11A* donors which targeted the N termini. The error bars represent the s.e.m. of *n* = 3 biologically independent experiments. **c**, Long-term stability of transgene expression at *HSP90AA1* (left) and *ACTB* (right) post sorting on Day 3 after dCas9–SSAP knock-in. Variable sorting efficiencies led to different starting mKate^+^ rates (full time course in Extended Data Fig. 9). Source data

**Extended Data Fig. 8:**
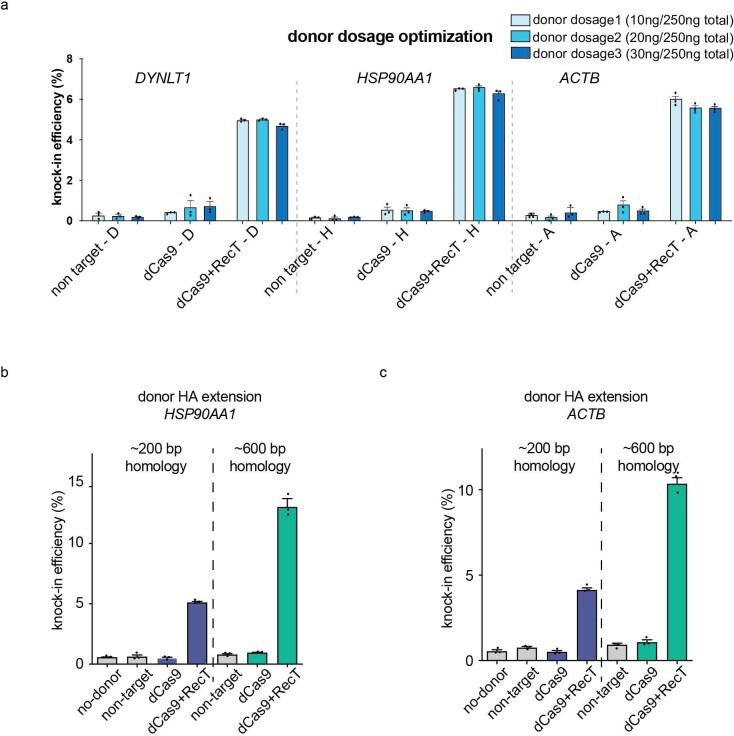
Optimization of donor dosages and homology arms of donor DNA. (**a**) Quantify genomic mKate knock-in efficiency at *DYNLT1*, *HSP90AA1*, *ACTB* loci for donor dosage optimization when using dCas9–SSAP editor. non target, non-target controls. Donor-HA lengths are 200 bp+200 bp for *DYNLT1*, 200 bp + 400 bp for *HSP90AA1*, 200 bp + 400 bp for *ACTB*. Quantify mKate knock-in efficiency at *HSP90AA1* (**b**) and *ACTB* (**c**) locus for donor homology arm (HA) optimization when using dCas9–SSAP editor. non target, non-target controls. Donor-HA lengths are 200 bp + 200 bp or 673 bp + 750 bp for *HSP90AA1*, 200 bp + 400 bp or 500 bp + 800 bp for *ACTB*. n = 3 biologically independent experiments. All results in this figure are from replicate experiments with error bars representing standard error of the mean (S.E.M.). Source data

We further optimized the dCas9–SSAP editor and tested its activities across a larger panel of genomic targets. We first examined whether adjustments to dosage could improve the level of editing efficiency (Fig. 4a and Extended Data Fig. 8a). When we increased the amount of SSAP-encoding plasmid, we observed higher editing efficiencies across all targets (Fig. 4a). This correlation further supported that the knock-in editing was driven by the SSAP. In contrast, increases in the amount of donor had negligible effects on the knock-in efficiency (Extended Data Fig. 8a), suggesting that the donor dosage was not a bottleneck in this setting. In addition to dosage optimization, we extended the donor-HA lengths and observed that further extension of the HAs helped to improve the knock-in efficiency, consistent with earlier results (Extended Data Fig. 8b,c).

Using these optimized parameters, we measured the level knock-in efficiency of dCas9–SSAP at seven endogenous loci (*DYNLT1*, *HSP90AA1*, *ACTB*, *BCAP31*, *HIST1H2BK*, *CLTA* and *RAB11A*; Fig. 4b). We included two loci (*CLTA* and *RAB11A*) where the knock-in tag was inserted as a direct fusion at the N termini of the endogenous proteins, complementing the 2A-peptide designs. Across all targets, dCas9–SSAP demonstrated efficiencies of up to about 20% without selection, which was comparable and sometimes moderately higher than the Cas9 references (Fig. 4b).

**Extended Data Fig. 9:**
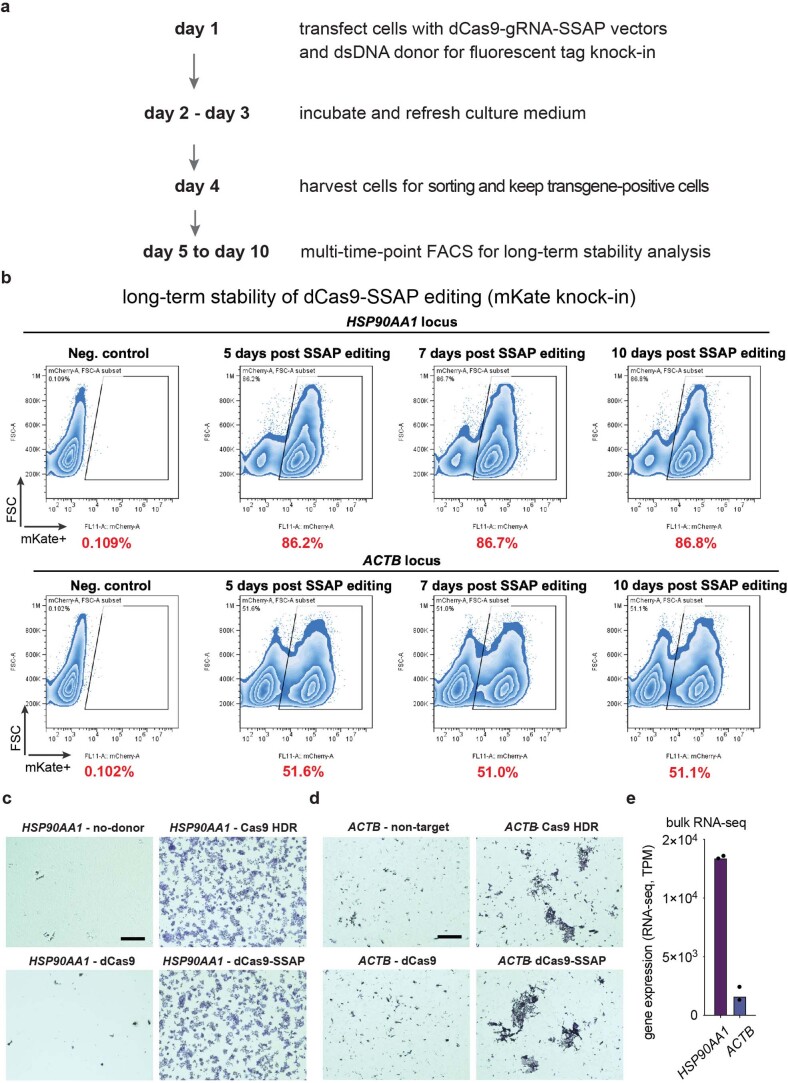
Validation the stability of on-target editing. (**a**) Workflow of the long-term time-course experiments to evaluate the editing outcome stability using dCas9–SSAP editor. (**b**) Flow cytometry analysis of knock-in gene editing at *HSP90AA1*, *ACTB* endogenous loci at different time points post delivery of dCas9–SSAP and donor DNA. (**c**, **d**) Representative crystal violet staining images for the on-target puromycin knock-in at *HSP90AA1* and *ACTB* locus. Scale bar = 500 um. The assay has been performed 3 times with similar results. (**e**) Quantify *HSP90AA1* and *ACTB* gene expression levels in HEK293T cells by bulk RNA-seq analysis, demonstrating notable higher levels of HSP90AA1 expression. This led to the better cell survival in the HSP90AA1 group compared with ACTB group. Data from 2 biologically independent experiments are presented. Source data

To ensure the stability of editing mediated by dCas9–SSAP over an extended time span, we next examined the durability of knock-in-transgene expression. We sorted mKate^+^ cells on Day 3 post transfection of dCas9–SSAP and donor DNA, and then checked whether the transgene maintained its expression beyond the three-day window at different genomic loci (Fig. 4c). Consistent with our sequencing results showing accurate on-target editing (Fig. 2), we observed that expression of the knock-in cassette was stable on Days 5, 7 and 10 post the delivery of dCas9–SSAP (Fig. 4c and Extended Data Fig. 9). The knock-in cell populations had distinct, steady transgene expression compared with the controls (Extended Data Fig. 9b). Thus, these data provided support for the utility of dCas9–SSAP for stable knock-in editing in mammalian cells.

**Fig. 5:**
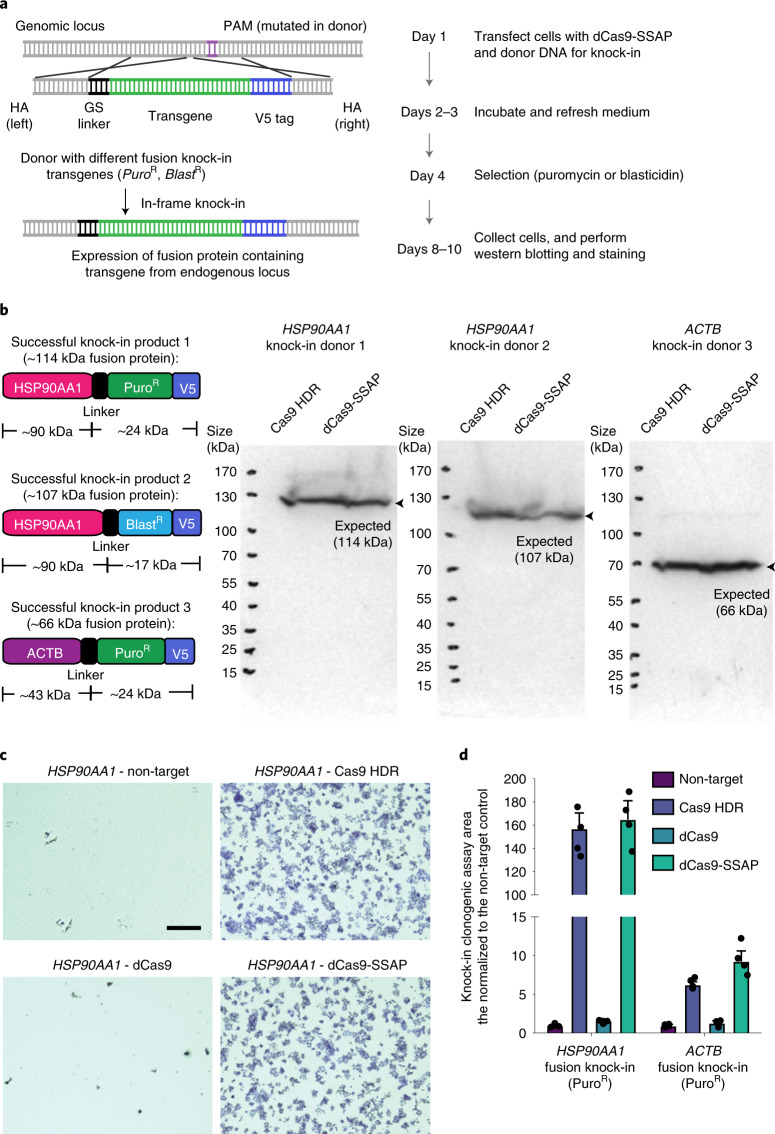
Validation of the dCas9–SSAP editor using protein functional assays. **a**, Design of the genomic puromycin- and blasticidin-resistance-cassette knock-in assay to validate functional on-target editing by dCas9–SSAP. **b**, Immunoblotting confirmation of the presence and sizes of on-target dCas9–SSAP knock-in products at the *HSP90AA1* and *ACTB* loci, performed with antibody to V5, which recognizes in-frame fusion with endogenous protein. Data are representative of *n* = 3 biologically independent experiments. Schematics (not to scale) of the knock-in proteins are shown (left). **c**,**d**, Validation and quantification of on-target knock-in using dCas9–SSAP via colony formation assays. Cells were selected by the knock-in resistance cassettes, stained with crystal violet (**c**) and quantified (**d**). Scale bar, 500 µm. The error bars represent the s.e.m. of *n* = 4 biologically independent experiments. Source data

Finally, we sought to functionally validate the ability of the dCas9–SSAP editor to insert diverse payloads at endogenous loci (Fig. 5a). Briefly, we constructed knock-in donors with selectable payloads (puromycin- and blasticidin-resistance cassettes) as fusion protein with endogenous genes (Fig. 5b, left). We examined the knock-in results from the dCas9–SSAP and Cas9-reference editors using western blotting. Immunoblotting confirmed the presence and correct sizes of the expected knock-in fusion proteins using dCas9–SSAP across targets (*HSP90AA1* and *ACTB*) and payloads (Fig. 5b). Furthermore, we quantified the relative knock-in efficiencies of the dCas9–SSAP and Cas9 methods using a functional assay (Fig. 5c and Extended Data Fig. 9c–e). We employed short-HA donors to insert a resistance cassette into endogenous loci and applied puromycin to select the knock-in cells. Colony formation assays validated that the dCas9–SSAP editor performed reliably using this protein-function readout (Fig. 5c).

### Dependence of dCas9–SSAP on endogenous pathways

**Fig. 6:**
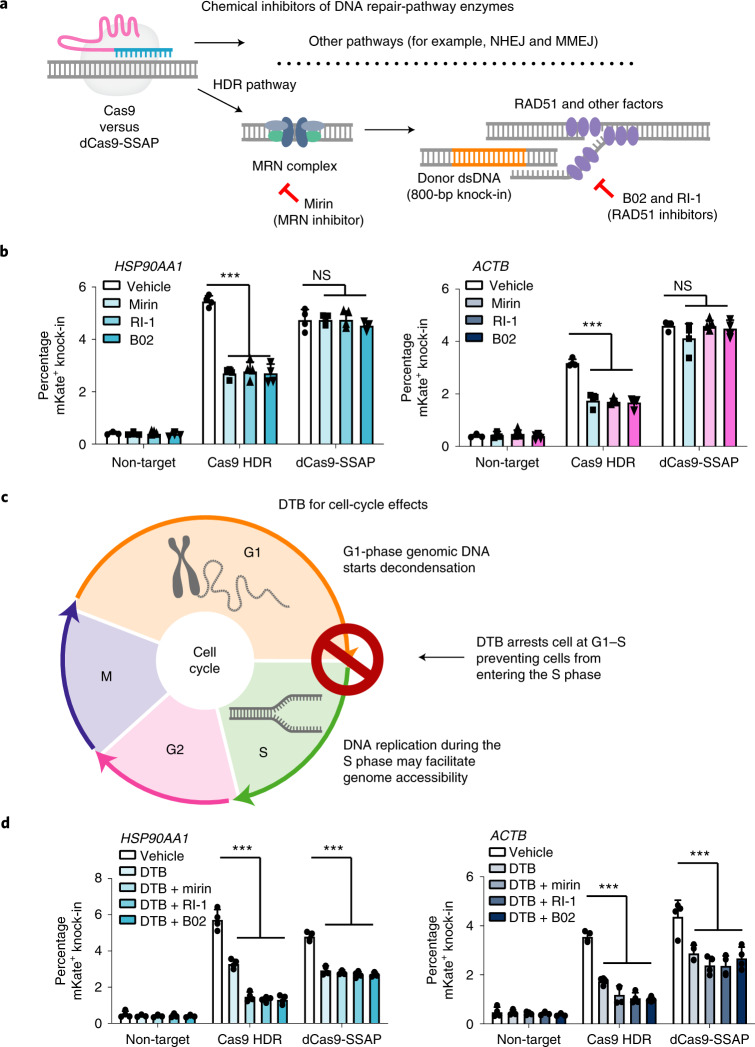
Chemical perturbation to probe the editing mechanism of dCas9–SSAP. **a**–**d**, Schematics showing experiment designs to measure the influence of endogenous DNA repair enzymes and cell cycle progression on dCas9-SSAP editing (**a**,**c**). Gene-editing efficiency (**b**,**d**) of the dCas9–SSAP editor when treated with DNA repair-pathway inhibitors (mirin, RI-1 and B02) without (**a**,**b**) or with (**c**,**d**) cell-cycle synchronization (DTB). The donors were the same as those used in Fig. 1. **b**,**d**, Statistical analyses were from the *t*-test results with a false-detection rate of 1% from the two-stage step-up method of Benjamini, Krieger and Yekutieli. The error bars represent the s.e.m. of *n* = 4 biologically independent experiments. Statistical analyses and comparisons were performed using a Student’s *t*-test; ***P < 0.001 and NS, not significant. Source data

Recall our model that dCas9–SSAP performs gene editing without DNA cleavage. To better understand the nature of dCas9–SSAP editing, we used three orthogonal chemical perturbations to examine its dependency on endogenous pathways (Fig. 6).

First, we perturbed enzymes in DSB-repair pathways during dCas9–SSAP and canonical Cas9 editing and compared the effects (Fig. 6a). In Cas9-mediated knock-in, the recognition of DSBs by the Mre11–Rad50–Nbs1 (MRN) complex is a necessary step for downstream HDR^12^. We leveraged mirin, a potent inhibitor of DSB repair that has been shown to prevent MRN complex formation, ATM activation and Mre11 exonuclease activity^56^. We treated cells with mirin and determined the level of editing efficiency of the dCas9–SSAP and Cas9-reference editors on these cells. Across all targets, we observed that the dCas9–SSAP efficiencies were nearly unaffected by the mirin treatment and essentially the same as the vehicle-treated groups (Fig. 6b). However, as expected, the Cas9 methods demonstrated substantially reduced levels of editing efficiency under the mirin treatment (Fig. 6b).

Second, we investigated the dependence of dCas9–SSAP editing on core enzymes of the HDR pathway. We used two small-molecule inhibitors of the RAD51 protein, RI-1 and B02, to block this rate-limiting step in HDR^57,58^. Our data showed that RAD51 inhibition significantly reduced the efficiency of Cas9 editing at all genomic targets but did not have a significant effect on dCas9–SSAP editing (Fig. 6b, RI-1 and B02). These two repair-modulating experiments generated consistent results: dCas9–SSAP showed significantly less sensitivity to the perturbations of several endogenous repair enzymes than Cas9 references. They suggest that the mechanism of the dCas9–SSAP editor differs from Cas9 editing.

Third, we investigated how cell cycling affects the dCas9–SSAP editor. Cell cycling has been shown to facilitate the accessibility of mammalian genomes^59^. More specifically, genome replication (during the S phase) may provide a favourable environment for dCas9 to unwind DNA and allow SSAP-mediated recombination (Fig. 6c). To test this, we synchronized cells at the G1–S boundary using double thymidine blockage (DTB)^60,61^. The DTB treatment indeed reduced the efficiency of dCas9–SSAP editing (Fig. 6d). Nonetheless, when we combined mirin, RI-1 or B02 with DTB treatment, dCas9–SSAP maintained higher levels of editing efficiency than the Cas9 references (Fig. 6d).

Together, our data supported the hypothetical mechanism of dCas9–SSAP editing: RNA-guided dCas9 binds to genomic targets and makes them accessible to the SSAP, and SSAP promotes homology-directed insertion without the requirement for a DNA break (Fig. 1a). Deeper understanding of this process will require further investigation—for example, biophysical analysis of the dCas9–SSAP editor or additional assays to modulate genome accessibility and repair pathways. Such insights could help to further develop dCas9 editing approaches.

### Minimization of dCas9–SSAP for convenient delivery

**Fig. 7:**
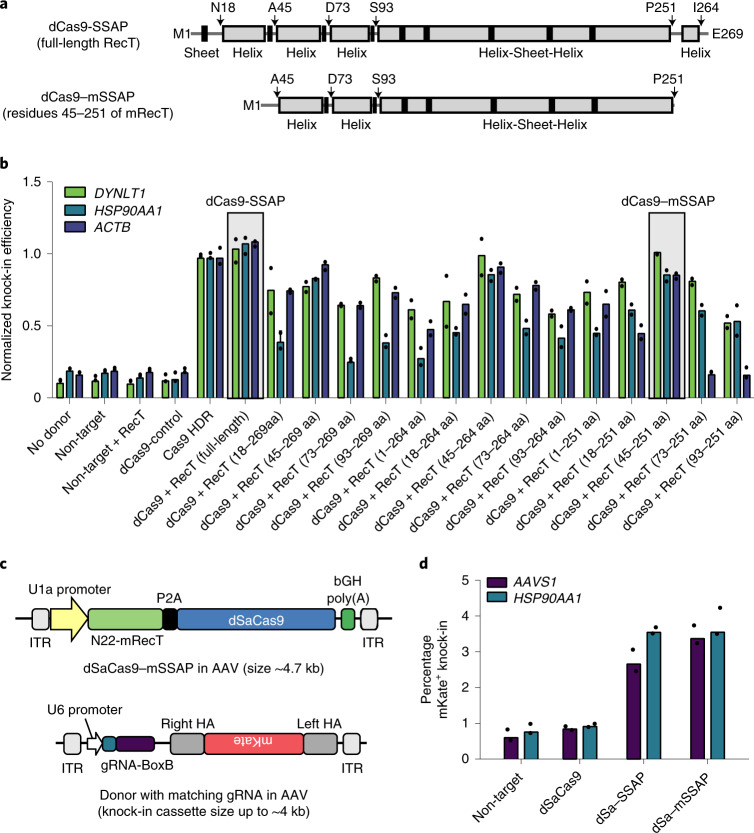
Minimization of dCas9–SSAP as a compact editing tool for convenient delivery. **a**, Predicted secondary structure and priming sites for constructing truncated EcRecT protein. **b**, Relative knock-in efficiencies of the truncation designs. All groups were normalized to the Cas9 references (individually for each target). aa, amino-acid residue. **c**,**d**, Schematic of the dSaCas9–mSSAP system in AAV construct using the compact SaCas9 (**c**; not shown to scale) and its knock-in (800 bp) efficiencies at *AAVS1* and *HSP90AA1* using in vitro delivery of AAV2 vectors carrying the original and minimized dSaCas9–SSAP editors in HEK293T cells (**d**). **b**,**d**, Data are for *n* = 2 biologically independent experiments. Source data

**Extended Data Fig. 10:**
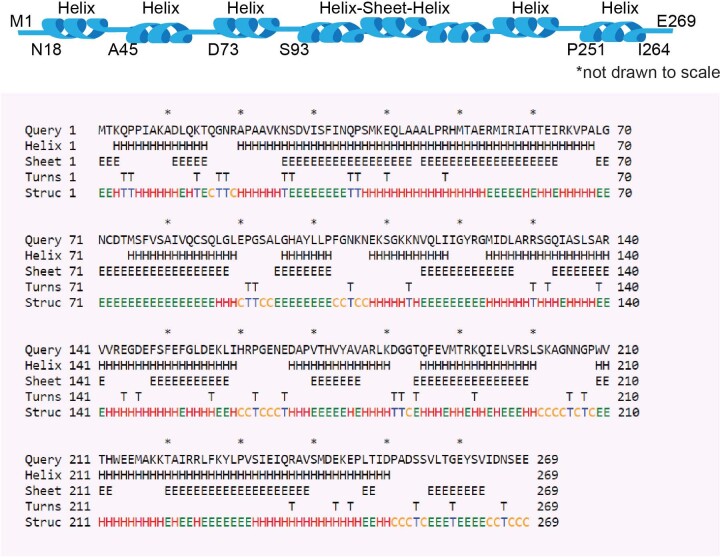
Schematic showing the RecT protein secondary structure predicted using online tool (CFSSP, see Methods). The prediction results (secondary structure visualized at top, alignment at bottom) formed the basis for developing a truncated functional RecT variant.

Finally, to optimize the dCas9–SSAP editor for future applications, we sought to develop a minimal version compatible with the size limitations of viral vectors such as AAV^20,21^. We designed 14 different truncated RecT SSAPs based on secondary-structure predictions (Fig. 7a and Extended Data Fig. 10) and tested their gene-editing activities alongside the full-length controls. We identified a short RecT variant (around 200 amino acids in length) that had comparable efficiencies to the original full-length RecT-based design (Fig. 7b).

We then integrated this short SSAP with the more compact SaCas9 system^62^ and the smaller N22-BoxB aptamer^63^ to build a minimal-functional dSaCas9–mSSAP editor (Fig. 7c). This allowed us to fit the dSaCas9–mSSAP into a single AAV and employ a ≤4 kb donor AAV for long-sequence editing (Fig. 7c). We tested the dSaCas9–mSSAP editor via delivery of AAV2 particles and confirmed that it had comparable efficiencies to the full-length version in HEK293T cells (Fig. 7d). This design, while needing further in vivo validation, could provide a convenient option for delivering the dCas9–SSAP editor.

## Discussion

Here we report the development of a dCas9–SSAP editor, which harmonizes the RNA-guided programmability of CRISPR with the SSAP activity of phage RecT. This dCas9–SSAP editor enables long-sequence editing with minimal DNA damage and errors. It provides research and therapeutic possibilities for addressing some of the currently intractable diseases involving large disease-causing variants, delivering therapeutic genes in vivo or minimizing undesirable modifications during gene editing^19,21^. Compared with other editing methods that depend on single-strand nicks or DSBs, dCas9–SSAP facilitates homology-mediated transgene insertion via non-cutting dCas9s. There are several remaining questions and development directions for this editing tool. First, it will be exciting to further understand the mechanism of dCas9–SSAP editing in mammalian cells. Based on our model and perturbation experiments, one possibility is that the strand-invasion activity of SSAP could help initiate the pairing of homologous sequences between the donor and accessible genomic DNA, followed by endogenous DNA synthesis and then resolution and integration of the knock-in sequences during DNA replication (which help explain the cell-cycle effects). Although dCas9–SSAP may be less dependent on certain endogenous repair enzymes, this process will still involve DNA repair or synthesis machinery. Thus, additional work—for example, systematic knock-out of repair enzymes—could help understand such involvement. Mining additional SSAPs from nature could also enhance the editing rates. Other delivery options, such as using mRNA or ribonucleoprotein, could help boost the dCas9–SSAP editor for broader applications, including primary-cell engineering using electroporation. Overall, this efficient low-error technology offers a complementary approach to existing CRISPR editing tools for long-sequence engineering.

## Methods

### Plasmid construction

Human codon-optimized DNA fragments were ordered from Genescript, Genewiz and IDT DNA. The fragments encoding the recombination enzymes were Gibson assembled into backbones (Addgene, plasmid 61423) using the NEBuilder HiFi DNA assembly master mix (New England BioLabs). The amino-acid sequence for these SSAP can be found in Supplementary Table 1. All sgRNAs were inserted into backbones (p-dCas9–SSAP-MS2-BB_BbsI and p-dsaCas9-SSAP-BoxB-BB_BsaI) using Golden Gate cloning. The dCas9–SSAP plasmids bearing sequences recognized by the restriction enzymes BbsI (dSpCas9) and BsaI (dSaCas9) as gRNA backbones were sequence-verified (Eton and Genewiz). The sgRNA sequences used in this research can be found in Supplementary Table 2. The list of all dCas9–SSAP plasmids are in Supplementary Table 3 and will be deposited to Addgene for open access.

### Cell culture

HEK293T, HeLa, HepG2 and U2OS cells were obtained from the American Type Culture Collection and maintained in Dulbecco’s Modified Eagle’s Medium (DMEM; Life Technologies) with 10% fetal bovine serum (FBS; BenchMark), 100 U ml^−1^ penicillin and 100 µg ml^−1^ streptomycin (Life Technologies) at 37 °C with 5% CO_2_. The hESC (H9) cells were maintained in mTeSR1 medium (StemCell Technologies) at 37 °C with 5% CO_2_. Culture plates were pre-coated with Matrigel (Corning) 12 h before use. The plates were washed three times with PBS before seeding with cells. The Rho kinase inhibitor Y27632 (10 µM; Sigma) was added for the first 24 h after each passage. The culture medium was changed every 24 h.

### Transfection

HEK293T, HeLa, HepG2 and U2OS cells were seeded into 96-well plates (Corning) at a density of 3 × 10^4^ cells per well 12 h before transfection with 250 ng total DNA per well. The cells were transfected using Lipofectamine 3000 (Life Technologies) following the manufacturer’s instructions when the cells were at approximately 70% confluency. Briefly, we used 250 ng total DNA and 0.4 µl Lipofectamine 3000 reagent, mixed with 10 µl Opti-MEM, per well. For 250 ng DNA, we used 160 ng of dCas9-gRNA plasmids (for the double sgRNA design, we used equal amounts of the two gRNA plasmids; that is, 80 ng each), 60 ng pMCP-RecT or GFP control plasmid (Addgene, 64539) and 30 ng of PCR template DNA (the primer sequences are listed in Supplementary Table 4 and the template sequences are listed in Supplementary Notes). The cells were analysed after 3 d using FACS. The step-by-step dCas9–SSAP gene-editing protocol can be found at *Protocol Exchange*^64^.

### Electroporation

For the transfection of hESC (H9) cells, a P3 primary cell 4D-NucleofectorTM X kit S (Lonza) was used following the manufacturer’s protocol. Briefly, the hESC (H9) cells were resuspended in Accutase (Innovative Cell Technology) and washed twice with PBS before electroporation. For each reaction, 3 × 10^5^ cells were nucleofected with 4 µg total DNA mixed in 20 µl electroporation buffer using the DC100 Nucleofector program. For 4 µg DNA, we used 2.6 µg of the dCas9–SSAP gRNA plasmids, 1 µg of pMCP-RecT or GFP control plasmid and 0.4 µg of PCR template DNA (the primer sequences are listed in Supplementary Table 4 and the template sequence are listed in Supplementary Notes). After electroporation, the cells were seeded into 12-well plates with 1 ml mTeSR1 medium containing 10 µM Y27632. The culture medium was changed every 24 h. After 4 d, the cells were analysed using a CytoFLEX flow cytometer (Beckman Coulter; Stanford Stem Cell FACS Core).

### FACS

The efficiency of the mKate knock-in was analysed on a CytoFLEX flow cytometer. The cells were washed twice with PBS 72 h after transfection or 96 h after electroporation and dissociated with TrypLE express enzyme (Thermo Fisher Scientific) or Accutase. The cell suspension was then transferred to a 96-well U-bottom plate (Thermo Fisher Scientific) and centrifuged at 300*g* for 5 min. The supernatant was aspirated, the pelleted cells were resuspended in 50 µl of 4% FBS in PBS and the cells were analysed on a CytoFLEX flow cytometer within 30 min following preparation.

### Long time-point mKate fluorescence monitoring

To monitor the editing stability over time, we sorted the mKate^+^ cells 48 h after transfection using an Aria II SORP system and maintained these cells in DMEM medium with 10% FBS, 100 U ml^−1^ penicillin and 100 µg ml^−1^ streptomycin. The mKate ratio was analysed at different time points as mentioned earlier.

### Western blotting

On-target knock-in of the GS-puromycin and blasticidin-V5 tag was verified by western blotting. The samples were collected 72 h after transfection and the proteins were extracted. Monoclonal antibody to the V5 tag (1:2,000; Thermo Scientific, R960-25) was used to detect the on-target editing product.

### Crystal violet assay

The efficiency of the GS-puromycin-V5 tag knock-in was analysed using a crystal violet assay. Cells in a 24-well plate were dissociated 72 h after transfection with TrypLE express enzyme and transferred to a six-well plate. The cells were maintained in DMEM medium with 10% FBS, 100 U ml^−1^ penicillin, 100 µg ml^−1^ streptomycin and 0.5 µg ml^−1^ puromycin (InvivoGen, ant-pr-1) at 37 °C with 5% CO_2_ for another 3–5 d. The crystal violet assay was performed once the puromycin selection had completed. The medium was removed and the plates were washed with PBS. The PBS was then removed, 2 ml of a mixture of 4% paraformaldehyde and 0.5% crystal violet was added (Sigma, C6158-50G) and the plates were left at room temperature for 30 min. The crystal violet mixture was carefully removed and the samples were washed with PBS. The plates were left to dry at room temperature and imaged using a Keyence microscope. The clones were quantified using the ImageJ software.

### Sanger sequencing analysis of knock-in junctions

HEK293T cells were harvested 72 h after transfection. The genomic DNA was extracted using QuickExtract DNA extraction solution (Biosearch Technologies) following the manufacturer’s instructions. The target genomic region was amplified using specific primers that bound outside of the HAs of the donor template. The primers used for the Sanger and NGS analyses are listed in Supplementary Table 4. The PCR products were purified using a Monarch PCR & DNA cleanup kit (New England BioLabs). The purified product (80–100 ng) was sent for Sanger sequencing with target-specific primers (EtonBio or Genewiz). The Sanger trace was analysed using the SnapGene software.

### Treatment with HR and cell-cycle inhibitor

For different inhibitor assays, the cells were pre-treated with mirin (Sigma, M9948-5MG; 25 µM), B02 (Sigma, SML0364; 10 µM) or RI-1 (Sigma, 553514-10MG-M; 1 µM) for 16 h. For the cell-cycle arrest experiment, the cells were pre-treated with thymidine (Sigma, T9250-1G, 2 mM) for 18 h, the thymidine was removed, the cells were cultured in normal DMEM media with 10% FBS without thymidine for 9 h and thymidine was added to the cells (final concentration of 2 mM) for a second round of 18 h. For the DTB–mirin/RI-1/B02 groups, mirin (25 µM), B02 (10 µM) or RI-1 (1 µM) were added to the cells with the second treatment round with thymidine (2 mM). After the inhibitor and thymidine treatment, the cells were transfected using Lipofectamine 3000 following the manufacturer’s instructions. The cells were analysed on a CytoFLEX flow cytometer 3 d later.

### NGS library preparation

Genomic DNA was extracted from cells 72 h after transfection using QuickExtract DNA extraction solution following the manufacturer’s instructions; 200 ng of genomic DNA was used for the NGS library preparation. Genes of interest were amplified using specific primers (primers are listed in Supplementary Table 4) for the first-round PCR reaction. Illumina adaptors and index barcodes were added with a second round of PCR using the primers listed in Supplementary Table 4. The PCR products were purified by gel electrophoresis on a 2% E-gel using a Monarch DNA gel extraction kit (New England BioLabs). The purified products were quantified using a Qubit dsDNA HS assay kit (Thermo Fisher) and sequenced on an Illumina MiSeq system using paired-end PE300 kits. All sequencing data were deposited to the NCBI Sequence Read Archive database under the accession code PRJNA683925.

### TOPO cloning experiment

A total of 250 ng genomic DNA was used for the TOPO cloning experiment. The knock-in events were amplified using specific TA colony primers targeting the *DYNLT1* or *HSP90AA1* locus (the primers are listed in Supplementary Table 4) using Phusion flash high-fidelity PCR master mix (Thermo Scientific, F-548L). The PCR products were purified using a gel extraction kit (New England BioLabs, T1020L) following the manufacturer’s instructions. A poly(A) tail was added to the purified products using Taq polymerase (New England BioLabs, M0273S) with incubation at 72 °C for 30 min. The TOPO cloning reaction was set up and the transformation was performed following the manufacturer’s instructions (Thermo Scientific, K457501). The plates were sent for rolling-circle amplification/colony sequencing using the M13F (5′-GTAAAACGACGGCCAG-3′) and M13R (5′-CAGGAAACAGCTATGAC-3′) universal Sanger sequencing primers. The sequence results were analysed using the SnapGene software.

### High-throughput sequencing data analysis

Processed (demultiplexed, trimmed and merged) sequencing reads were analysed to determine the editing outcomes using CRISPPResso2 by aligning the sequenced amplicons to the reference and expected HDR amplicons. The quantification window was increased to 10 bp surrounding the expected cut site to better capture diverse editing outcomes but substitutions were ignored to avoid the inclusion of sequencing errors. Only reads containing no mismatches to the expected amplicon were considered for HDR quantification; reads containing indels that partially matched the expected amplicons were included in the overall reported indel frequency.

### Insertion-site mapping and analysis

We used a process that was previously developed (GIS-seq) and adapted for the genome-wide, unbiased off-target analysis of mKate knock-in following the similar protocol in our previous study^31,65,66^. Briefly, we harvested the HEK293T cells 3 d after transfection. The genomic DNA was size-selected using a DNAdvance genomic DNA kit (A48705, Beckman Coulter) to avoid template contamination in the following step. The purified genomic DNA (400 ng) was fragmented to an average of 500 bp using NEB fragmentase, ligated with adaptors and size-selected using a NEBNext ultra II FS DNA library prep kit following the manufacturer’s instructions. Following two rounds of nested anchored PCR to amplify the target DNA (from the end of the knock-in sequence to the ligated adaptor sequence), a size-selection purification following the NEBNext Ultra II FS DNA library Prep kit protocol was performed. The libraries were sequenced using Illumina Miseq V3 PE600 kits. The sequencing data were analysed to determine off-target insertion events with custom analysis code.

### Statistics and reproducibility

Unless otherwise stated, all statistical analyses and comparisons were performed using a Student’s *t*-test, with a 1% false-discovery rate using the two-stage step-up method of Benjamini, Krieger and Yekutieli. The reproducibility, sample sizes and, where appropriate, statistical analyses are described in the figure legends.

### Reporting Summary

Further information on research design is available in the Nature Research Reporting Summary linked to this article.

## Online content

Any methods, additional references, Nature Research reporting summaries, source data, extended data, supplementary information, acknowledgements, peer review information; details of author contributions and competing interests; and statements of data and code availability are available at 10.1038/s41556-021-00836-1.

## Supplementary information

Supplementary Information Supplementary notes and references.

Reporting Summary

Supplementary Table 1 Supplementary Table 1. Sequences of all SSAPs tested in this study. Supplementary Table 2. Sequences of the gRNAs used in this study. Guides starting with sp indicate SpCas9 gRNA targets and guides starting with dsp indicate dSpCas9 gRNA targets. Supplementary Table 3. List of plasmids (all of which will be available at Addgene with a plasmid ID). Supplementary Table 4. Sequences of the primers used for donor DNA (HDR template) generation, targeted sequencing and NGS assays. All NGS adaptor sequences are shown in red.

## Data Availability

All NGS data, including data from the targeted genomic loci sequencing and on/off-target analysis have been deposited to the NCBI Sequence Read Archive database under the accession code PRJNA683925. All other data supporting the findings of this study are available from the corresponding author on reasonable request. Source data are provided with this paper.

## Source data

Source Data Fig. 1 Statistical source data.
Source Data Fig. 1 Unprocessed images.
Source Data Fig. 2 Statistical source data.
Source Data Fig. 3 Statistical source data.
Source Data Fig. 4 Statistical source data.
Source Data Fig. 5 Statistical source data.
Source Data Fig. 5 Unprocessed images and unprocessed western blots.
Source Data Fig. 6 Statistical source data.
Source Data Fig. 7 Statistical source data.
Source Data Extended Data Fig. 2 Statistical source data.
Source Data Extended Data Fig. 4 Statistical source data.
Source Data Extended Data Fig. 5 Statistical source data.
Source Data Extended Data Fig. 7 Statistical source data.
Source Data Extended Data Fig. 8 Statistical source data.
Source Data Extended Data Fig. 9 Statistical source data.
Source Data Extended Data Fig. 9 Unprocessed images.

## Code availability

Customized scripts for data analysis have been deposited and are available at GitHub under the Cong Laboratory repository (https://github.com/cong-lab).
