# Supplementary material for: dCas9-based gene editing for cleavage-free genomic knock-in of long sequences
Source: Nat Cell Biol. 2022 Feb 10;24(2):268–78. doi: 10.1038/s41556-021-00836-1 (PMC8843813; doi:10.1038/s41556-021-00836-1)
Supplement: Supplementary file 1 — Supplementary notes and references. [file 41556_2021_836_MOESM1_ESM.pdf]

nature portfolio

https://doi.org/10.1038/s41556-021-00836-1

Supplementary information

dCas9-based gene editing for cleavage-free genomic knock-in of long sequences

In the format provided by the authors and unedited

Supplementary Information

dCas9-based gene editing for cleavage-free genomic knock-in of long sequences

Supplementary Notes

Supplementary References

Supplementary Notes

SSAP mining process

For initial SSAP screening, we identified the three major family of phage recombination enzymes *(27)* from Bacteriophage lambda, *E. coli* Rac prophage, and bacteriophage T7, and extracted the primary enzyme sequences as listed in supplementary sequences. For RecT-like SSAP mining. RefSeq non-redundant protein database was downloaded from NCBI on October 29, 2019. We systematically searched the NCBI non-redundant sequence database for RecT homologs. After identifying a large set of 2,071 candidates, we built phylogenetic trees and selected representative candidates up to 300-aa (to avoid large proteins that are less portable) to refine the hits, and obtain a final list of 16 SSAPs (**Extended Data Fig. 2**). Overall, the SSAP candidates have significant heterogeneity, while retaining conserved regions that have been previously suggested to be important for their biochemical activities *(46-48)*.

Donor design test comparing Cas9 HDR, Cas9 MMEJ, and dCas9-SSAP

As shown in **Fig. 3b-c**, we tested the new editor with different donor DNA designs. We considered three major types of donor DNAs with different homology arm (HA) length designs. Specifically, we synthesized: 1) HDR donors bearing long HAs (>=100bp), a standard format for long-sequence engineering and transgene knock-in; 2) MMEJ donors with typically short HAs (<= 50bp), which have been shown to improve editing efficiencies for DSB-mediated knock-in*(54, 66-69)*; 3) NHEJ donors without HAs (0bp), which could help gauge the levels of donor integration due to Cas9-induced DSBs *(14, 15, 66)*. Our results from these tests revealed two characteristics of dCas9-SSAP that are distinct from Cas9 gene-editing.

Firstly, for the NHEJ donors without any HAs (highlighted box in **Fig. 3c**), we observed knock-in cassette expression when using Cas9 editor but not for the dCas9-SSAP editor (**Fig.**

3c). This is consistent with previous reports that Cas9-mediated DSBs could induce NHEJ-mediated donor DNA insertion *(14, 15)*, but this integration is minimal when using the non-cutting dCas9-SSAP (**Fig. 3c**, dCas9-SSAP with NHEJ 0bp donor). Secondly, dCas9-SSAP benefited from successively longer HA within the donor, regardless of whether the HAs are for HDR-type or MMEJ-type, in contrast to Cas9 editor that showed a boost of knock-in efficiencies when using the MMEJ donors (**Fig. 3c**, HDR and MMEJ donors). This is consistent with the assumption that the enhancing effect when using MMEJ donors is dependent on Cas9 cleavage of target genomic sites. Further, while the focus of this work is long-sequence engineering, we also tested dCas9-SSAP for shorter sequence editing (**Extended Data Fig. 6**) and observed precise knock-in of 16-bp sequence into *EMX1* locus in human HEK293T cells. In summary, dCas9-SSAP editing becomes most efficient when using HDR donors, and longer homology arms in general make editing efficiency higher.

Detailed notes for all construct and donor sequences

MCP-EXTEN-RecT-SV40 NLS

ATGGCTTCAAACTTTACTCAGTTCGTGCTCGTGGACAATGGTGGGACAGGGGATGTGACA
GTGGCTCCTTCTAATTTCGCTAATGGGGTGGCAGAGTGGATCAGCTCCAACTCACGGAGC
CAGGCCTACAAGGTGACATGCAGCGTCAGGCAGTCTAGTGCCCAGAAGAGAAAGTATACC
ATCAAGGTGGAGGTCCCCAAAGTGGCTACCCAGACAGTGGGCGGAGTCGAACTGCCTGT
CGCCGCTTGGAGGTCCTACCTGAACATGGAGCTCACTATCCCAATTTTCGCTACCAATTCT
GACTGTGAACTCATCGTGAAGGCAATGCAGGGGCTCCTCAAAGACGGTAATCCTATCCCTT
CCGCCATCGCCGCTAACTCAGGTATCTACAGCGCTTCCGGAGGATCTAGCGGAGGCTCCT
CTGGCTCTGAGACACCTGGCACAAGCGAGAGCGCAACACCTGAAAGCAGCGGGGGCAGC
AGCGGGGGGTCAGGAGGATCCATGACAAAACAACCTCCAATAGCAAAAGCTGATCTGCAG

AAAACCCAGGGCAATAGAGCCCCTGCTGCTGTGAAGAATAGTGACGTGATTAGCTTCATCA

ACCAGCCTAGCATGAAGGAGCAGCTGGCCGCCGCCCTGCCTCGCCACATGACCGCAGAG

CGGATGATTCGGATCGCTACAACCGAGATCAGAAAAGTGCCCGCTCTGGGGAACTGTGAT

ACCATGTCTTTCGTGAGCGCCATTGTGCAGTGTAGCCAGCTGGGCCTGGAGCCCGGAAGT

GCCCTGGGCCACGCCTACCTGCTGCCCTTCGGCAACAAGAACGAGAAATCCGGTAAAAAG

AACGTGCAGCTGATCATCGGTTATAGAGGTATGATCGATCTGGCTCGGCGGTCCGGCCAA

ATCGCCAGCCTGTCTGCCAGGGTGGTGAGAGAGGGCGACGAGTTCAGCTTTGAGTTTGGC

CTGGACGAAAAGTTGATCCATCGGCCCGGAGAGAACGAGGACGCCCCTGTCACCCACGT

GTATGCCGTGGCTAGACTGAAGGACGGCGGTACACAGTTCGAAGTTATGACCCGGAAGCA

GATCGAACTGGTGAGGTCCCTGTCCAAGGCCGGCAATAACGGGCCCTGGGTGACCCACT

GGGAAGAAATGGCCAAGAAAACCGCCATTAGACGCCTGTTCAAGTACCTGCCCGTGTCCA

TTGAGATTCAGAGGGCCGTGTCTATGGATGAGAAAGAGCCCCTGACCATCGACCCTGCTG

ACAGCAGCGTGCTGACCGGCGAGTACTCTGTGATTGACAATTCTGAGGAGTCTGGTGGTT

CTCCTAAGAAAAAGAGGAAGGTGTGA

N22-EXTEN-RecT-SV40 NLS

ATGGGTAATGCTCGGACCCGGCGAAGAGAGAGGCGGGCTGAGAAGCAGGCACAGTGGAA

GGCTGCAAACAGCGCTTCCGGAGGATCTAGCGGAGGCTCCTCTGGCTCTGAGACACCTG

GCACAAGCGAGAGCGCAACACCTGAAAGCAGCGGGGGCAGCAGCGGGGGGTCAGGAGG

ATCCATGACAAAACAACCTCCAATAGCAAAAGCTGATCTGCAGAAAACCCAGGGCAATAGA

GCCCCTGCTGCTGTGAAGAATAGTGACGTGATTAGCTTCATCAACCAGCCTAGCATGAAGG

AGCAGCTGGCCGCCGCCCTGCCTCGCCACATGACCGCAGAGCGGATGATTCGGATCGCT

ACAACCGAGATCAGAAAAGTGCCCGCTCTGGGGAACTGTGATACCATGTCTTTCGTGAGC

GCCATTGTGCAGTGTAGCCAGCTGGGCCTGGAGCCCGGAAGTGCCCTGGGCCACGCCTA

CCTGCTGCCCTTCGGCAACAAGAACGAGAAATCCGGTAAAAAGAACGTGCAGCTGATCAT

CGGTTATAGAGGTATGATCGATCTGGCTCGGCGGTCCGGCCAAATCGCCAGCCTGTCTGC
CAGGGTGGTGAGAGAGGGCGACGAGTTCAGCTTTGAGTTTGGCCTGGACGAAAAGTTGAT
CCATCGGCCCGGAGAGAACGAGGACGCCCCTGTCACCCACGTGTATGCCGTGGCTAGAC
TGAAGGACGGCGGTACACAGTTCGAAGTTATGACCCGGAAGCAGATCGAACTGGTGAGGT
CCCTGTCCAAGGCCGGCAATAACGGGCCCTGGGTGACCCACTGGGAAGAAATGGCCAAG
AAAACCGCCATTAGACGCCTGTTCAAGTACCTGCCCGTGTCCATTGAGATTCAGAGGGCC
GTGTCTATGGATGAGAAAGAGCCCCTGACCATCGACCCTGCTGACAGCAGCGTGCTGACC
GGCGAGTACTCTGTGATTGACAATTCTGAGGAGTCTGGTGGTTCTCCTAAGAAAAAGAGGA
AGGTGTGA

Donor DNA (HDR template) sequences

Annotations of the replaced or inserter editing sequences are detailed below with each of the donor DNAs. Unless otherwise noted, when different homology arms are used in the study, we used primers listed in **Table S2** to obtain donor DNAs with different homology arm lengths.

DYNLT1 P2A-mKate knock-in HDR template sequence

Left Homology Arm-Insertion Sequence-Right Homology Arm

(Underlined are the inserted mKate fluorescent protein sequence, the proceeding non-underlined part is the P2A peptide sequence)

AGTGACCTGTGTAATTATGCAGAAGAATGGAGCTGGATTACACACAGCAAGTTCCTGCTTC
TGGGACAGCTCTACTGACGGTATGATTTTCATTCATGTTTGTGAAGTTTTGTTGTGTGAAAT
ATATGACTGGAAGTTTCCTATCTTTGAATGCAATGCATGTTTATCACCTTTTAAAACATTTAA
TAATAGACTTGCCAAGGTTCTTTGTGTAGCATAGAGATGGGTACTTGAATGTTGGCCTTATT
GTGAGTAAAACGTCGTCCCCCAGCTTTCCCTGCCGTAAATGCTGCTCTCTTCCCTCCCGCA

GGGAGCTGCACTGTGCGATGGGAGAATAAGACCATGTACTGCATCGTCAGTGCCTTCGGA
CTGTCTATTGGAAGCGGAGCTACTAACTTCAGCCTGCTGAAGCAGGCTGGAGACGTGGAG
GAGAACCCTGGACCTGCCACCGTGAGCGAGCTGATTAAGGAGAACATGCACATGAAGCTG
TACATGGAGGGCACCGTGAACAACCACCACTTCAAGTGCACATCCGAGGGCGAAGGCAAG
CCCTACGAGGGCACCCAGACCATGAGAATCAAGGCGGTCGAGGGCGGCCCTCTCCCCTT
CGCCTTCGACATCCTGGCTACCAGCTTCATGTACGGCAGCAAAACCTTCATCAACCACACC
CAGGGCATCCCCGACTTCTTTAAGCAGTCCTTCCCCGAGGGCTTCACATGGGAGAGAGTC
ACCACATACGAAGATGGGGGCGTGCTGACCGCTACCCAGGACACCAGCCTCCAGGACGG
CTGCCTCATCTACAACGTCAAGATCAGAGGGGTGAACTTCCCATCCAACGGCCCTGTGAT
GCAGAAGAAAACACTCGGCTGGGAGGCCTCCACCGAGACACTGTACCCCGCTGACGGCG
GCCTGGAAGGCAGAGCCGACATGGCCCTGAAGCTCGTGGGCGGGGGCCACCTGATCTGC
AACCTTAAGACCACATACAGATCCAAGAAACCCGCTAAGAACCTCAAGATGCCCGGCGTCT
ACTATGTGGACAGGAGACTGGAAAGAATCAAGGAGGCCGACAAAGAGACATACGTCGAGC
AGCACGAGGTGGCTGTGGCCAGATACTGCGACCTCCCTAGCAAACTGGGGCACAAACTTA
ATTCCTAACCAGCTGTCCtGCCTATGGCCTTTCTCCTTTTGTCTCTAGTTCATCCTCTAACCA
CCAGCCATGAATTCAGTGAACTCTTTTCTCATTCTCTTTGTTTTGTGGCACTTTCACAATGTA
GAGGAAAAAACCAAATGACCGCACTGTGATGTGAATGGCACCGAAGTCAGATGAGTATCC
CTGTAGGTCACCTGCAGCCTGCGTTGCCACTTGTCTTAACTCTGAATATTTCATTTCAAAGG
TGCTAAAATCTGAAATCTGCTAGTGTGAAACTTGCTCTACTCTCTGAAATGATTCAAATACA
CTAATTTTCCATACTTTATACTTTTGTTAGAATAAATTATTCAAATCTAAAGTCTGTTGTGTTC
TTCATAGTCTGCATAGTATCATAAACG

HSP90AA1 P2A-mKate knock-in HDR template sequence

Left Homology Arm-Insertion Sequence-Right Homology Arm

(Underlined are the inserted mKate fluorescent protein sequence, the proceeding non-underlined part is the P2A peptide sequence)

GGCTGGACAGCAAACATGGAGAGAATCATGAAAGCTCAAGCCCTAAGAGACAACTCAACA
ATGGGTTACATGGCAGCAAAGAAACACCTGGAGATAAACCCTGACCATTCCATTATTGAGA
CCTTAAGGCAAAAGGCAGAGGCTGATAAGAACGACAAGTCTGTGAAGGATCTGGTCATCTT
GCTTTATGAAACTGCGCTCCTGTCTTCTGGCTTCAGTCTGGAAGATCCCCAGACACATGCT
AACAGGATCTACAGGATGATCAAACTTGGTCTGGGTAAGCCTTATACTATGTAATGTTAAAA
AGAAAATAAACACACGTGACATTGAAGAAAATGGTGAACTTTCAGTTATCCAAACTTGGAGC
ACCTTGTCCTGCTTGCTGCTTGGAGGTATTAAAGTATGTTTTTTTTAGGGATAAGTAAGGTC
TTACAAGAGCAAAGAAATGAAATTGAGACTCATATGTCCTGTAATACTGTCTTGAAAGCAGA
TAGAAACCAAGAGTATTACCCTAATAGCTGGCTTTAAGAAATCTTTGTAATATGAGGATTTTA
TTTTGGAAACAGGTATTGATGAAGATGACCCTACTGCTGATGATACCAGTGCTGCTGTAAC
TGAAGAAATGCCACCCCTTGAAGGAGATGACGACACATCACGCATGGAAGAAGTAGACGG
AAGCGGAGCTACTAACTTCAGCCTGCTGAAGCAGGCTGGAGACGTGGAGGAGAACCCTG
GACCTGTGAGCGAGCTGATTAAGGAGAACATGCACATGAAGCTGTACATGGAGGGCACCG
TGAACAACCACCACTTCAAGTGCACATCCGAGGGCGAAGGCAAGCCCTACGAGGGCACCC
AGACCATGAGAATCAAGGCGGTCGAGGGCGGCCCTCTCCCCTTCGCCTTCGACATCCTGG
CTACCAGCTTCATGTACGGCAGCAAAACCTTCATCAACCACACCCAGGGCATCCCCGACTT
CTTTAAGCAGTCCTTCCCCGAGGGCTTCACATGGGAGAGAGTCACCACATACGAAGATGG
GGGCGTGCTGACCGCTACCCAGGACACCAGCCTCCAGGACGGCTGCCTCATCTACAACG
TCAAGATCAGAGGGGTGAACTTCCCATCCAACGGCCCTGTGATGCAGAAGAAAACACTCG
GCTGGGAGGCCTCCACCGAGACACTGTACCCCGCTGACGGCGGCCTGGAAGGCAGAGCC
GACATGGCCCTGAAGCTCGTGGGCGGGGGCCACCTGATCTGCAACCTTAAGACCACATAC
AGATCCAAGAAACCCGCTAAGAACCTCAAGATGCCCGGCGTCTACTATGTGGACAGGAGA

CTGGAAAGAATCAAGGAGGCCGACAAAGAGACATACGTCGAGCAGCACGAGGTGGCTGT
GGCCAGATACTGCGACCTCCCTAGCAAACTGGGGCACAAACTTAATTCCTAAATCTGTGGC
TGAGGGATGACTTACCTGTTCAGTACTCTACAATTCCTCTGATAATATATTTTCAAGGATGTT
TTTCTTTATTTTTGTTAATATTAAAAAGTCTGTATGGCATGACAACTACTTTAAGGGGAAGAT
AAGATTTCTGTCTACTAAGTGATGCTGTGATACCTTAGGCACTAAAGCAGAGCTAGTAATGC
TTTTTGAGTTTCATGTTGGTTTATTTTCACAGATTGGGGTAACGTGCACTGTAAGACGTATG
TAACATGATGTTAACTTTGTGGTCTAAAGTGTTTAGCTGTCAAGCCGGATGCCTAAGTAGAC
CAAATCTTGTTATTGAAGTGTTCTGAGCTGTATCTTGATGTTTAGAAAAGTATTCGTTACATC
TTGTAGGATCTACTTTTTGAACTTTTCATTCCCTGTAGTTGACAATTCTGCATGTACTAGTCC
TCTAGAAATAGGTTAAACTGAAGCAACTTGATGGAAGGATCTCTCCACAGGGCTTGTTTTC
CAAAGAAAAGTATTGTTTGGAGGAGCAAAGTTAAAAGCCTACCTAAGCATATCGTAAAGCT
GTTCAAAAATAACTCAGACCCAGTCTTGTGGATGGAAATGTAGTGCTCGAGTCACATTCTG
CTTAAAGTTGTAACAAATACAGATGAGTTAAAAGATATTGTGTGACAGTGTCTTATTTAGGG
GGAAAGGGGAGTATCTGGATGACAGTTAGTGCCAAAATGTAAAACATGAGGCGCTAGCAG
GAGAT

OCT4 P2A-mKate knock-in HDR template sequence

Left Homology Arm-Insertion Sequence-Right Homology Arm

(Underlined are the inserted mKate fluorescent protein sequence, the proceeding non-underlined part is the P2A peptide sequence)

GCGACTATGCACAACGAGAGGATTTTGAGGCTGCTGGGTCTCCTTTCTCAGGGGGACCAG
TGTCCTTTCCTCTGGCCCCAGGGCCCCATTTTGGTACCCCAGGCTATGGGAGCCCTCACT
TCACTGCACTGTACTCCTCGGTCCCTTTCCCTGAGGGGAAGCCTTTCCCCCTGTCTCCGT
CACCACTCTGGGCTCTCCCATGCATTCAAATGGAAGCGGAGCTACTAACTTCAGCCTGCTG

AAGCAGGCTGGAGACGTGGAGGAGAACCCTGGACCTGCCACCATGGTGAGCGAGCTGAT

TAAGGAGAACATGCACATGAAGCTGTACATGGAGGGCACCGTGAACAACCACCACTTCAA

GTGCACATCCGAGGGCGAAGGCAAGCCCTACGAGGGCACCCAGACCATGAGAATCAAGG

CGGTCGAGGGCGGCCCTCTCCCCTTCGCCTTCGACATCCTGGCTACCAGCTTCATGTACG

GCAGCAAAACCTTCATCAACCACACCCAGGGCATCCCCGACTTCTTTAAGCAGTCCTTCCC

CGAGGGCTTCACATGGGAGAGAGTCACCACATACGAAGATGGGGGCGTGCTGACCGCTA

CCCAGGACACCAGCCTCCAGGACGGCTGCCTCATCTACAACGTCAAGATCAGAGGGGTGA

ACTTCCCATCCAACGGCCCTGTGATGCAGAAGAAAACACTCGGCTGGGAGGCCTCCACCG

AGACACTGTACCCCGCTGACGGCGGCCTGGAAGGCAGAGCCGACATGGCCCTGAAGCTC

GTGGGCGGGGGCCACCTGATCTGCAACCTTAAGACCACATACAGATCCAAGAAACCCGCT

AAGAACCTCAAGATGCCCGGCGTCTACTATGTGGACAGGAGACTGGAAAGAATCAAGGAG

GCCGACAAAGAGACATACGTCGAGCAGCACGAGGTGGCTGTGGCCAGATACTGCGACCT

CCCTAGCAAACTGGGGCACAAACTTAATTCCTAATGACTAGGAATGGGGGACAGGGGGAG

GGGAGGAGCTAGGGAAAGAAAACCTGGAGTTTGTGCCAGGGTTTTTGGGATTAAGTTCTT

CATTCACTAAGGAAGGAATTGGGAACACAAAGGGTGGGGGCAGGGGAGTTTGGGGCAAC

TGGTTGGAGGGAAGGTGAAGTTCAATGATGCTCTTGATTTTAATCCCACATCATGTATCACT

TTTTTCTTAAATAAAGAAGCCTGGGACACAGTAGATAGACACACTT

ACTB P2A-mKate knock-in HDR template sequence

Left Homology Arm-Insertion Sequence-Right Homology Arm

(Underlined are the inserted mKate fluorescent protein sequence, the proceeding non-underlined part is the P2A peptide sequence)

CGGCTCTGCCTGACATGAGGGTTACCCCTCGGGGCTGTGCTGTGGAAGCTAAGTCCTGCC

CTCATTTCCCTCTCAGGCATGGAGTCCTGTGGCATCCACGAAACTACCTTCAACTCCATCA

TGAAGTGTGACGTGGACATCCGCAAAGACCTGTACGCCAACACAGTGCTGTCTGGCGGCA

CCACCATGTACCCTGGCATTGCCGACAGGATGCAGAAGGAGATCACTGCCCTGGCACCCA

GCACAATGAAGATCAAGGTGGGTGTCTTTCCTGCCTGAGCTGACCTGGGCAGGTCGGCTG

TGGGGTCCTGTGGTGTGTGGGGAGCTGTCACATCCAGGGTCCTCACTGCCTGTCCCCTTC

CCTCCTCAGATCATTGCTCCTCCTGAGCGCAAGTACTCCGTGTGGATCGGCGGCTCCATC

CTGGCCTCGCTGTCCACCTTCCAGCAGATGTGGATCAGCAAGCAGGAGTATGACGAGTCC

GGCCCCTCCATCGTCCACCGCAAGTGTTTCGGAAGCGGAGCTACTAACTTCAGCCTGCTG

AAGCAGGCTGGAGACGTGGAGGAGAACCCTGGACCTGTGAGCGAGCTGATTAAGGAGAA

CATGCACATGAAGCTGTACATGGAGGGCACCGTGAACAACCACCACTTCAAGTGCACATC

CGAGGGCGAAGGCAAGCCCTACGAGGGCACCCAGACCATGAGAATCAAGGCGGTCGAGG

GCGGCCCTCTCCCCTTCGCCTTCGACATCCTGGCTACCAGCTTCATGTACGGCAGCAAAA

CCTTCATCAACCACACCCAGGGCATCCCCGACTTCTTTAAGCAGTCCTTCCCCGAGGGCTT

CACATGGGAGAGAGTCACCACATACGAAGATGGGGGCGTGCTGACCGCTACCCAGGACA

CCAGCCTCCAGGACGGCTGCCTCATCTACAACGTCAAGATCAGAGGGGTGAACTTCCCAT

CCAACGGCCCTGTGATGCAGAAGAAAACACTCGGCTGGGAGGCCTCCACCGAGACACTGT

ACCCCGCTGACGGCGGCCTGGAAGGCAGAGCCGACATGGCCCTGAAGCTCGTGGGCGG

GGGCCACCTGATCTGCAACCTTAAGACCACATACAGATCCAAGAAACCCGCTAAGAACCTC

AAGATGCCCGGCGTCTACTATGTGGACAGGAGACTGGAAAGAATCAAGGAGGCCGACAAA

GAGACATACGTCGAGCAGCACGAGGTGGCTGTGGCCAGATACTGCGACCTCCCTAGCAAA

CTGGGGCACAAACTTAATTCCTAATAGGCGGACTATGACTTAGTTGCGTTACACCCTTTCTT

GACAAAACCTAACTTGCGCAGAAAACAAGATGAGATTGGCATGGCTTTATTTGTTTTTTTTG

TTTTGTTTTGGTTTTTTTTTTTTTTTTGGCTTGACTCAGGATTTAAAAACTGGAACGGTGAAG

GTGACAGCAGTCGGTTGGAGCGAGCATCCCCCAAAGTTCACAATGTGGCCGAGGACTTTG

ATTGCACATTGTTGTTTTTTTAATAGTCATTCCAAATATGAGATGCGTTGTTACAGGAAGTCC

CTTGCCATCCTAAAAGCCACCCCACTTCTCTCTAAGGAGAATGGCCCAGTCCTCTCCCAAG

TCCACACAGGGGAGGTGATAGCATTGCTTTCGTGTAAATTATGTAATGCAAAATTTTTTTAA

TCTTCGCCTTAATACTTTTTTATTTTGTTTTATTTTGAATGATGAGCCTTCGTGCCCCCCCTT

CCCCCTTTTTTGTCCCCCAACTTGAGATGTATGAAGGCTTTTGGTCTCCCTGGGAGTGGGT

GGAGGCAGCCAGGGCTTACCTGTACACTGACTTGAGACCAGTTGAATAAAAGTGCACACC

TTAAAAATGAGGCCAAGTGTGACTTTGTGGTGTGGCTGGGTTGGGGGCAGCAGAGGGTGA

ACCCTGCAGGAGGGTGAACCCTGCAAAAGGGTGGGGCAGTGGGGGCCAACTTGTCCTTA

CCCAGAGTGCAGGTGTGTGGAGATCCCTCCTGCCTTGACATTGAGCAGCCTTAGAGGGTG

GGGGAGGCTCAGGGGTCAGGTCTCTGTTC

EMX1 HDR template sequence

Left Homology Arm-Insertion/Replacement Sequence-Right Homology Arm

(Underlined are the inserted BsrGI restriction site, i.e. TGTACA)

CATTCTGCCTCTCTGTATGGAAAAGAGCATGGGGCTGGCCCGTGGGGTGGTGTCCACTTT

AGGCCCTGTGGGAGATCATGGGAACCCACGCAGTGGGTCATAGGCTCTCTCATTTACTAC

TCACATCCACTCTGTGAAGAAGCGATTATGATCTCTCCTCTAGAAACTCGTAGAGTCCCAT

GTCTGCCGGCTTCCAGAGCCTGCACTCCTCCACCTTGGCTTGGCTTTGCTGGGGCTAGAG

GAGCTAGGATGCACAGCAGCTCTGTGACCCTTTGTTTGAGAGGAACAGGAAAACCACCCT

TCTCTCTGGCCCACTGTGTCCTCTTCCTGCCCTGCCATCCCCTTCTGTGAATGTTAGACCC

ATGGGAGCAGCTGGTCAGAGGGGACCCCGGCCTGGGGCCCCTAACCCTATGTAGCCTCA

GTCTTCCCATCAGGCTCTCAGCTCAGCCTGAGTGTTGAGGCCCCAGTGGCTGCTCTGGGG

GCCTCCTGAGTTTCTCATCTGTGCCCCTCCCTCCCTGGCCCAGGTGAAGGTGTGGTTCCA

GAACCGGAGGACAAAGTACAAACGGCAGAAGCTGGAGGAGGAAGGGCCTGAGTCCGAGC

AGAAGAAGAAGGGCTCCCATCACATCAACCGGTGGCGCATTGCCACGAAGCAGGCCAATG

GGGAGGACATCGATGTCACCTCCAATGACTCGGATGTACACGGTCTGCAACCACAAACCC

ACGAGGGCAGAGTGCTGCTTGCTGCTGGCCAGGCCCCTGCGTGGGCCCAAGCTGGACTC

TGGCCACTCCCTGGCCAGGCTTTGGGGAGGCCTGGAGTCATGGCCCCACAGGGCTTGAA
GCCCGGGGCCGCCATTGACAGAGGGACAAGCAATGGGCTGGCTGAGGCCTGGGACCACT
TGGCCTTCTCCTCGGAGAGCCTGCCTGCCTGGGCGGGCCCGCCCGCCACCGCAGCCTCC
CAGCTGCTCTCCGTGTCTCCAATCTCCCTTTTGTTTTGATGCATTTCTGTTTTAATTTATTTT
CCAGGCACCACTGTAGTTTAGTGATCCCCAGTGTCCCCCTTCCCTATGGGAATAATAAAAG
TCTCTCTCTTAATGACACGGGCATCCAGCTCCAGCCCCAGAGCCTGGGGTGGTAGATTCC
GGCTCTGAGGGCCAGTGGGGGCTGGTAGAGCAAACGCGTTCAGGGCCTGGGAGCCTGG
GGTGGGGTACTGGTGGAGGGGGTCAAGGGTAATTCATTAACTCCTCTCTTTTGTTGGGGG
ACCCTGGTCTCTACCTCCAGCTCCACAGCAGGAGAAACAGGCTAGACATAGGGAAGGGCC
ATCCTGTATCTTGAGGGAGGACAGGCCCAGGTCTTTCTTAACGTATTGAGAGGTGGGAATC
AGGCCCAGGTAGTTCAATGGG

DYNLT1 mKate-T2A-EGFP HDR template

Left Homology Arm-mKate-T2Alinker-EGFP-Right Homology Arm

(Underlined are the inserted **mKate/EGFP** fluorescent protein sequence, with the connecting non-underlined **T2A peptide** sequence)

TGCCGTAAATGCTGCTCTCTTCCCTCCCGCAGGGAGCTGCACTGTGCGATGGGAGAATAA
GACCATGTACTGCATCGTCAGTGCCTTCGGACTGTCTATTGGAAGCGGAGCTACTAACTTC
AGCCTGCTGAAGCAGGCTGGAGACGTGGAGGAGAACCCTGGACCTGCCACCATGGTGAG
CGAGCTGATTAAGGAGAACATGCACATGAAGCTGTACATGGAGGGCACCGTGAACAACCA
CCACTTCAAGTGCACATCCGAGGGCGAAGGCAAGCCCTACGAGGGCACCCAGACCATGA
GAATCAAGGCGGTCGAGGGCGGCCCTCTCCCCTTCGCCTTCGACATCCTGGCTACCAGCT
TCATGTACGGCAGCAAAACCTTCATCAACCACACCCAGGGCATCCCCGACTTCTTTAAGCA
GTCCTTCCCCGAGGGCTTCACATGGGAGAGAGTCACCACATACGAAGATGGGGGCGTGCT

GACCGCTACCCAGGACACCAGCCTCCAGGACGGCTGCCTCATCTACAACGTCAAGATCAG
AGGGGTGAACTTCCCATCCAACGGCCCTGTGATGCAGAAGAAAACACTCGGCTGGGAGGC
CTCCACCGAGACACTGTACCCCGCTGACGGCGGCCTGGAAGGCAGAGCCGACATGGCCC
TGAAGCTCGTGGGCGGGGGCCACCTGATCTGCAACCTTAAGACCACATACAGATCCAAGA
AACCCGCTAAGAACCTCAAGATGCCCGGCGTCTACTATGTGGACAGGAGACTGGAAAGAA
TCAAGGAGGCCGACAAAGAGACATACGTCGAGCAGCACGAGGTGGCTGTGGCCAGATAC
TGCGACCTCCCTAGCAAACTGGGGCACAAACTTAATTCCGCTAGCGGCAGTGGAGAGGGC
AGAGGAAGTCTGCTAACATGCGGTGACGTCGAGGAGAATCCTGGCCCAGGTGGTTCTGCC
GGTGGCTCCGGTTCTGGCTCCAGCGGTGGCAGCTCTGGTGCGTCCGGCACGGGTACTGC
GGGTGGCACTGGCAGCGGTTCCGGTACTGGCTCTGGCGTGAGCAAGGGCGAGGAGCTGT
TCACCGGGGTGGTGCCCATCCTGGTCGAGCTGGACGGCGACGTAAACGGCCACAAGTTC
AGCGTGTCCGGCGAGGGCGAGGGCGATGCCACCTACGGCAAGCTGACCCTGAAGTTCAT
CTGCACCACCGGCAAGCTGCCCGTGCCCTGGCCCACCCTCGTGACCACCCTGACCTACG
GCGTGCAGTGCTTCAGCCGCTACCCCGACCACATGAAGCAGCACGACTTCTTCAAGTCCG
CCATGCCCGAAGGCTACGTCCAGGAGCGCACCATCTTCTTCAAGGACGACGGCAACTACA
AGACCCGCGCCGAGGTGAAGTTCGAGGGCGACACCCTGGTGAACCGCATCGAGCTGAAG
GGCATCGACTTCAAGGAGGACGGCAACATCCTGGGGCACAAGCTGGAGTACAACTACAAC
AGCCACAACGTCTATATCATGGCCGACAAGCAGAAGAACGGCATCAAGGTGAACTTCAAG
ATCCGCCACAACATCGAGGACGGCAGCGTGCAGCTCGCCGACCACTACCAGCAGAACAC
CCCCATCGGCGACGGCCCCGTGCTGCTGCCCGACAACCACTACCTGAGCACCCAGTCCG
CCCTGAGCAAAGACCCCAACGAGAAGCGCGATCACATGGTCCTGCTGGAGTTCGTGACCG
CCGCCGGGATCACTCTCGGCATGGACGAGCTGTACAAGTGACCAGCTGTCCtGCCTATGG
CCTTTCTCCTTTTGTCTCTAGTTCATCCTCTAACCACCAGCCATGAATTCAGTGAACTCTTTT
CTCATTCTCTTTGTTTTGTGGCACTTTCACAATGTAGAGGAAAAACCAAATGACCGCACTG
TGATGTGAATGGCACCGAAGTCAGATGAGTATCCCTGTAGGTCACCTGCAGCCTGCGTTG
CCACTTGTCTT

HSP90AA1 mKate-T2A-EGFP HDR template

Left Homology Arm-mKate-T2Alinker-EGFP-Right Homology Arm

(Underlined are the inserted **mKate/EGFP** fluorescent protein sequence, with the connecting non-underlined **T2A peptide** sequence)

TACTGTCTTGAAAGCAGATAGAAACCAAGAGTATTACCCTAATAGCTGGCTTTAAGAAATCT
TTGTAATATGAGGATTTTATTTTGGAAACAGGTATTGATGAAGATGACCCTACTGCTGATGA
TACCAGTGCTGCTGTAACTGAAGAAATGCCACCCCTTGAAGGAGATGACGACACATCACG
CATGGAAGAAGTAGACGGAAGCGGAGCTACTAACTTCAGCCTGCTGAAGCAGGCTGGAGA
CGTGGAGGAGAACCCTGGACCTGCCACCATGGTGAGCGAGCTGATTAAGGAGAACATGCA
CATGAAGCTGTACATGGAGGGCACCGTGAACAACCACCACTTCAAGTGCACATCCGAGGG
CGAAGGCAAGCCCTACGAGGGCACCCAGACCATGAGAATCAAGGCGGTCGAGGGCGGCC
CTCTCCCCTTCGCCTTCGACATCCTGGCTACCAGCTTCATGTACGGCAGCAAAACCTTCAT
CAACCACACCCAGGGCATCCCCGACTTCTTTAAGCAGTCCTTCCCCGAGGGCTTCACATG
GGAGAGAGTCACCACATACGAAGATGGGGGCGTGCTGACCGCTACCCAGGACACCAGCC
TCCAGGACGGCTGCCTCATCTACAACGTCAAGATCAGAGGGGTGAACTTCCCATCCAACG
GCCCTGTGATGCAGAAGAAAACACTCGGCTGGGAGGCCTCCACCGAGACACTGTACCCC
GCTGACGGCGGCCTGGAAGGCAGAGCCGACATGGCCCTGAAGCTCGTGGGCGGGGGCC
ACCTGATCTGCAACCTTAAGACCACATACAGATCCAAGAAACCCGCTAAGAACCTCAAGAT
GCCCGGCGTCTACTATGTGGACAGGAGACTGGAAAGAATCAAGGAGGCCGACAAAGAGA
CATACGTCGAGCAGCACGAGGTGGCTGTGGCCAGATACTGCGACCTCCCTAGCAAACTGG
GGCACAAACTTAATTCCGCTAGCGGCAGTGGAGAGGGCAGAGGAAGTCTGCTAACATGCG
GTGACGTCGAGGAGAATCCTGGCCCAGGTGGTTCTGCCGGTGGCTCCGGTTCTGGCTCC
AGCGGTGGCAGCTCTGGTGCGTCCGGCACGGGTACTGCGGGTGGCACTGGCAGCGGTTC
CGGTACTGGCTCTGGCGTGAGCAAGGGCGAGGAGCTGTTCACCGGGGTGGTGCCCATCC

TGGTCGAGCTGGACGGCGACGTAAACGGCCACAAGTTCAGCGTGTCCGGCGAGGGCGAG
GGCGATGCCACCTACGGCAAGCTGACCCTGAAGTTCATCTGCACCACCGGCAAGCTGCCC
GTGCCCTGGCCCACCCTCGTGACCACCCTGACCTACGGCGTGCAGTGCTTCAGCCGCTAC
CCCGACCACATGAAGCAGCACGACTTCTTCAAGTCCGCCATGCCCGAAGGCTACGTCCAG
GAGCGCACCATCTTCTTCAAGGACGACGGCAACTACAAGACCCGCGCCGAGGTGAAGTTC
GAGGGCGACACCCTGGTGAACCGCATCGAGCTGAAGGGCATCGACTTCAAGGAGGACGG
CAACATCCTGGGGCACAAGCTGGAGTACAACTACAACAGCCACAACGTCTATATCATGGCC
GACAAGCAGAAGAACGGCATCAAGGTGAACTTCAAGATCCGCCACAACATCGAGGACGGC
AGCGTGCAGCTCGCCGACCACTACCAGCAGAACACCCCCATCGGCGACGGCCCCGTGCT
GCTGCCCGACAACCACTACCTGAGCACCCAGTCCGCCCTGAGCAAAGACCCCAACGAGAA
GCGCGATCACATGGTCCTGCTGGAGTTCGTGACCGCCGCCGGGATCACTCTCGGCATGG
ACGAGCTGTACAAGTGAATCTGTGGCTGAGGGATGACTTACCTGTTCAGTACTCTACAATT
CCTCTGATAATATATTTTCAAGGATGTTTTTCTTTATTTTTGTTAATATTAAAAAGTCTGTATG
GCATGACAACTACTTTAAGGGGAAGATAAGATTTCTGTCTACTAAGTGATGCTGTGATACCT
TAGGCACTAAAGCAGAGCTAGTAATGCT

HIST1H2BK P2A-mKate knock-in HDR template sequence

Left Homology Arm-Insertion Sequence-Right Homology Arm

(Underlined are the inserted mKate fluorescent protein sequence, the proceeding non-underlined part is the P2A peptide sequence)

AGGCCATGGGAATCATGAACTCCTTCGTCAACGACATCTTCGAACGCATCGCGGGTGAGG
CTTCCCGCCTGGCGCATTACAACAAGCGCTCGACCATCACCTCCAGGGAGATCCAGACGG
CCGTGCGCCTGCTGCTGCCCGGGGAGTTGGCCAAGCACGCCGTGTCCGAGGGCACCAAG
GCCGTCACCAAGTACACCAGCGCTAAGAGATCTGGAAGCGGAGCTACTAACTTCAGCCTG
CTGAAGCAGGCTGGAGACGTGGAGGAGAACCCTGGACCTATGGTGAGCGAGCTGATTAA
GGAGAACATGCACATGAAGCTGTACATGGAGGGCACCGTGAACAACCACCACTTCAAGTG
CACATCCGAGGGCGAAGGCAAGCCCTACGAGGGCACCCAGACCATGAGAATCAAGGCGG
TCGAGGGCGGCCCTCTCCCCTTCGCCTTCGACATCCTGGCTACCAGCTTCATGTACGGCA
GCAAAACCTTCATCAACCACACCCAGGGCATCCCCGACTTCTTTAAGCAGTCCTTCCCCGA
GGGCTTCACATGGGAGAGAGTCACCACATACGAAGATGGGGGCGTGCTGACCGCTACCC
AGGACACCAGCCTCCAGGACGGCTGCCTCATCTACAACGTCAAGATCAGAGGGGTGAACT
TCCCATCCAACGGCCCTGTGATGCAGAAGAAAACACTCGGCTGGGAGGCCTCCACCGAGA
CACTGTACCCCGCTGACGGCGGCCTGGAAGGCAGAGCCGACATGGCCCTGAAGCTCGTG
GGCGGGGGCCACCTGATCTGCAACCTTAAGACCACATACAGATCCAAGAAACCCGCTAAG
AACCTCAAGATGCCCGGCGTCTACTATGTGGACAGGAGACTGGAAAGAATCAAGGAGGCC
GACAAAGAGACATACGTCGAGCAGCACGAGGTGGCTGTGGCCAGATACTGCGACCTCCCT
AGCAAACTGGGGCACAAACTTAATTCCTAATAAACTTGGAAAGTAAGCGTCTTAAAGCCCA
ACCCCAAAGGCTCTTTTAAGAGCCACTTAAATTATCGATATTAGAGCTGTAAACACGTGTTC
CTAATCCAGGCTTAAATTCGGGCGATCTTTTTAAAGGGATTATCATGATCTTATCACACTGA
GTAATGCATGCAGTGCTTGTAATCGAGTCTGGCTAAGGAGAAGCCACATACACTTTGTTAG
TACTGAGTGAGGAATATGGCGCCTAAAAAATTAGGGATGTGCAGGAAACTTGTGTTAACAG
AAAGTGCTTCCTGGCTCTTGGCGCGAAAATGGATACTTCCGTTGTGTCGAGTGCTGTGACT
TCCTGTTAGAACTTGTTGAAAGCCTATTGTGTCACGTGTACTTTCCACCATGTAATGGCGTT
CTAACGTGAG

BCAP31 P2A-mKate knock-in HDR template sequence

Left Homology Arm-Insertion Sequence-Right Homology Arm

(Underlined are the inserted mKate fluorescent protein sequence, the proceeding non-underlined part is the P2A peptide sequence)

AGTCCTCAGACTAGCTTGGGTGCACCAGGCCGCATCTGGAAGCCCAGGCGGTCTTGACGT
TGGGAGTGAGGGCCACATAAATACTCCTAACTTTGGACAGCTGGCCTCAGTGCTTGGTGG
CCCAAGTTTGGGCACCACAGCAGGTGGGGAGGAAGGGTCCAGCCTCAAAGGGGGCACCC
ATGCCCTCTGTATGGGGCAGGCTTGGCAGATGGGCTTCGAGGGCACCTGTCAAAGGTGTG
CAGAGTTGGGAAGGGTCTGATCAGGTCTTGAAGGGGACTGGATGCCTTGCTGTTCCCTGC
AGCCAGGACTCAGCCCCCAGTGAGGTCCTGGCAGTCCTCCCTGGCTGGCGTTAGGTCCA
GGGCTTTCCCTAGAGCGTGGGCCAGGGCTGACGCTCCCACCCTGGCAGGCCTTTGGGTG
CAGCTGGGGAGGGGGCCCCTTGTTCACTTGAATAGCTGTTGTTAGGAGAGAGGGGAACC
GAGGTGGACCTCTGGGGCATGGGGCTGGAGGTGGCAGGGGAGGAGTGGACCCGGCCAA
CCTACTGCTGTGGGATTTCTGTCCCTTTCCAGGCTGCAGTAGATGGTCCCATGGACAAGAA
GGAAGAGAGATCTGGAAGCGGAGCTACTAACTTCAGCCTGCTGAAGCAGGCTGGAGACGT
GGAGGAGAACCCTGGACCTATGGTGAGCGAGCTGATTAAGGAGAACATGCACATGAAGCT
GTACATGGAGGGCACCGTGAACAACCACCACTTCAAGTGCACATCCGAGGGCGAAGGCAA
GCCCTACGAGGGCACCCAGACCATGAGAATCAAGGCGGTCGAGGGCGGCCCTCTCCCCT
TCGCCTTCGACATCCTGGCTACCAGCTTCATGTACGGCAGCAAAACCTTCATCAACCACAC
CCAGGGCATCCCCGACTTCTTTAAGCAGTCCTTCCCCGAGGGCTTCACATGGGAGAGAGT
CACCACATACGAAGATGGGGGCGTGCTGACCGCTACCCAGGACACCAGCCTCCAGGACG
GCTGCCTCATCTACAACGTCAAGATCAGAGGGGTGAACTTCCCATCCAACGGCCCTGTGA
TGCAGAAGAAAACACTCGGCTGGGAGGCCTCCACCGAGACACTGTACCCCGCTGACGGC
GGCCTGGAAGGCAGAGCCGACATGGCCCTGAAGCTCGTGGGCGGGGGCCACCTGATCTG

CAACCTTAAGACCACATACAGATCCAAGAAACCCGCTAAGAACCTCAAGATGCCCGGCGTC
TACTATGTGGACAGGAGACTGGAAAGAATCAAGGAGGCCGACAAAGAGACATACGTCGAG
CAGCACGAGGTGGCTGTGGCCAGATACTGCGACCTCCCTAGCAAACTGGGGCACAAACTT
AATTCCTAATAAGGGCCTCCTTCCTCCCCTGCCTGCAGCTGGCTTCCACCTGGCACGTGC
CTGCTGCTTCCTGAGAGCCCGGCCTCTCCCTCCAGTACTTCTGTTTGTGCCCTTCTGCTTC
CCCCATTCCCTTCCACAGCTCATAGCTCGTCATCTCGGCCCTTGTCCACACTCTCCAAGCA
CATTACAGGGGACCTGATTGCTACACGTTCAGAATGCGTTTGCTGTCATCCTGCTTGGCCT
GGCCAGGCCTGGCACAGCCTTGGCTTCCACGCCTGAGCGTGGAGAGCACGAGTTAGTTG
TAGTCCGGCTTGCGGTGGGGCTGACTTCCTGTTGGTTTGAGCCCCTTTTTGTTTTGCCCTC
TGGGTGTTTTCTTTGGTCCCGCAGGAGGGTGGGTGGAGCAGGTGGACTGGAGTTTCTCTT
GAGGGCAATAAAAGTTGTCATGGTGTGTACGTGGTGCGGTGTGTGACTGCAGCTGTGGGG
AGGGCTCTGCTTTCTCTCCTGCCTGGAGCCCCCAAGCGGCCACAGCCCTTCAACCTGTTT
CCCCCACTGAAGGCCAGGCTCACACCTGTTCTCGAAGGCCTTGCCCTCTCTGTCCTCCTG
CTCCCCGGGCTGGGCCAGGCCTCGCTTCAGGGCCACCCGCCGAGTGTGTGTCCTGTCTG
CCTCCCTGGGTGTGGGCAGCTTGGGAGGCTCAGTAAACCGGCGGGGCTTCCGGGTAGAG
GACTCGCTTGCCTTCTCTGGGTTCC

CLTA mKate knock-in HDR template sequence

Left Homology Arm-Insertion Sequence-Right Homology Arm

(Underlined are the inserted mKate fluorescent protein sequence)

GTAAATCTATGGGCTCTAAGGGAGCAAATAATCCAGGCTGGCTGAAAGAATTAAATATTTTA
AATTTATAAAGCCAGGGACCCTCAAATCCATAAAAGGATTCAATCTTCTTCTTCTTTTTTTTT

AGGATTCAATCTTCTAATAATCCCGGGAGGGGGCGCAAGAGCAGGATTCTTGTTTCTATTT
TACGAATGAGGAAACTGAGGTTCAGAAAGCCTAAGTGGCATGCCTCTTCAGTAATCGGCCA
ATCGTCGCTGCCCTAACTTCACCTCACCTCCCAGTGCAGCACTAGAGTCCCCTCAGGGGT
GGCCGTCCCTGGTCAGTCCAGCTCCAGCAGACGGGGTGGGTAGGACGCTCCCTATCAGG
CAGCACTTCCGCCTCCCGGGGCCCGCGCAGCTCACCTCCCTCACCTCCCGCCCTACCCC
AGTCACGAGTTGTTTTAGGGGGACCGCCCCTCCACTTGCTGATTGGGTAGCTCCTGAACC
ATTGTTGTCCTCTGATTGGTTGTTCCCTTTTCGGCTCTGCAACACCGCCTAGACCGACCGG
ATACACGGGTAGGGCTTCCGCTTTACCCGTCTCCCTCCTGGCGCTTGTCCTCCTCTCCCA
GTCGGCACCACAGCGGTGGCTGCCGGGCGTGGTGTCGGTGGGTCGGTTGGTTTTTGTCT
CACCGTTGGTGTCCGTGCCGTTCAGTTGCCCGCCATGGCTGTGAGCGAGCTGATTAAGGA
GAACATGCACATGAAGCTGTACATGGAGGGCACCGTGAACAACCACCACTTCAAGTGCAC
ATCCGAGGGCGAAGGCAAGCCCTACGAGGGCACCCAGACCATGAGAATCAAGGCGGTCG
AGGGCGGCCCTCTCCCCTTCGCCTTCGACATCCTGGCTACCAGCTTCATGTACGGCAGCA
AAACCTTCATCAACCACACCCAGGGCATCCCCGACTTCTTTAAGCAGTCCTTCCCCGAGGG
CTTCACATGGGAGAGAGTCACCACATACGAAGATGGGGGCGTGCTGACCGCTACCCAGGA
CACCAGCCTCCAGGACGGCTGCCTCATCTACAACGTCAAGATCAGAGGGGTGAACTTCCC
ATCCAACGGCCCTGTGATGCAGAAGAAAACACTCGGCTGGGAGGCCTCCACCGAGACACT
GTACCCCGCTGACGGCGGCCTGGAAGGCAGAGCCGACATGGCCCTGAAGCTCGTGGGC
GGGGGCCACCTGATCTGCAACCTTAAGACCACATACAGATCCAAGAAACCCGCTAAGAAC
CTCAAGATGCCCGGCGTCTACTATGTGGACAGGAGACTGGAAAGAATCAAGGAGGCCGAC
AAAGAGACATACGTCGAGCAGCACGAGGTGGCTGTGGCCAGATACTGCGACCTCCCTAGC
AAACTGGGGCACAAACTTAATTCCGAGCTGGATCCGTTCGGCGCCCCTGCCGGCGCCCCT
GGCGGTCCCGCGCTGGGGAACGGAGTGGCCGGCGCCGGCGAAGAAGACCCGGCTGCG
GCCTTCTTGGCGCAGCAAGAGAGCGAGATTGCGGGCATCGAGAACGACGAGGCCTTCGC
CATCCTGGACGGCGGCGCCCCCGGGCCCCAGCCGCACGGCGAGCCGCCGGGGGGTCC
GGGTGAGAGTGCGGGCGCGTTTGGGGCGAGAGGACTTGTCTGGAAACTCGGTCCACAGT

GGGTCCGAGAGCTTCTGTGTGACTCGTGCTCCTTGCTGAATTAGGAGGTTAGGGAGCAGT

GCAAACAGGAAACGAGACCCTGGCCCGGTCTTTCAGAAACCTAGGCTCGAGAAGCCTGTT

CGGTTCTCAGCATGTTTGAGTGCTTCTGGGCGCGGGCGGAGCGAGAAAGCAAGTGTAGG

GTGGCAGGCTCCGGAGCCGGAAGAAGCCCGTTCAATTCAGCAACTTTTCATTAAGCATTTG

CTGTGCCTTTAGTCCGGTCTCTGAAGCAACCGCATTGGCGCAGTTTTTCCAGACTTATAAG

CTTATAAGTCTGAGCCGAGCACAGAACTCGTTA

RAB11A mKate knock-in HDR template sequence

Left Homology Arm-Insertion Sequence-Right Homology Arm

(Underlined are the inserted mKate fluorescent protein sequence)

GGAACCGCCACGCATGTGTAGCTGCCTTCGGCTGTCTAATCCTCAGAGAACCCCGCCCCC

ATCCACAAACCCACCACTCACAGGCGGTCCCGCCTGGTTCCAGCGAGCCGCTTCCGGCAC

GGTAGCTCGAGAAATGAGCAAGCGGCCACTAAGACTATGGTAGCTAGGAGTTCCAGGACT

CAGTTTCCCCTTTGAGCCTCCTTTAGCGACTAAAGCTTGAAGCCCCACGCATCTCGACTCT

CGCGCACACCGCCCTTGTTGGGCTCAGGGGCGGGGCGCCGCCCCCGGAAGTACTTCCCC

TTAAAGGCTGGGGCCTGCCGGAAATGGCGCAGCGGCAGGGAGGGGCTCTTCACCCAGTC

CGGCAGTTGAAGCTCGGCGCTCGGGTTACCCCTGCAGCGACGCCCCCTGGTCCCACAGA

TACCACTGCTGCTCCCGCCCTTTCGCTCCTCGGCCGCGCAATGGGCGTGAGCGAGCTGAT

TAAGGAGAACATGCACATGAAGCTGTACATGGAGGGCACCGTGAACAACCACCACTTCAA

GTGCACATCCGAGGGCGAAGGCAAGCCCTACGAGGGCACCCAGACCATGAGAATCAAGG

CGGTCGAGGGCGGCCCTCTCCCCTTCGCCTTCGACATCCTGGCTACCAGCTTCATGTACG

GCAGCAAAACCTTCATCAACCACACCCAGGGCATCCCCGACTTCTTTAAGCAGTCCTTCCC

CGAGGGCTTCACATGGGAGAGAGTCACCACATACGAAGATGGGGGCGTGCTGACCGCTA

CCCAGGACACCAGCCTCCAGGACGGCTGCCTCATCTACAACGTCAAGATCAGAGGGGTGA

ACTTCCCATCCAACGGCCCTGTGATGCAGAAGAAAACACTCGGCTGGGAGGCCTCCACCG

AGACACTGTACCCCGCTGACGGCGGCCTGGAAGGCAGAGCCGACATGGCCCTGAAGCTC

GTGGGCGGGGGCCACCTGATCTGCAACCTTAAGACCACATACAGATCCAAGAAACCCGCT

AAGAACCTCAAGATGCCCGGCGTCTACTATGTGGACAGGAGACTGGAAAGAATCAAGGAG

GCCGACAAAGAGACATACGTCGAGCAGCACGAGGTGGCTGTGGCCAGATACTGCGACCT

CCCTAGCAAACTGGGGCACAAACTTAATTCCACACGTGACGACGAGTACGACTACCTCTTT

AAAGGTGAGGCCATGGGCTCTCGCACTCTACACAGTCCTCGTTCGGGGACCCGGGCCACT

CCCGGTGGACCCTCGTGCCGGCCACCCCTGCACTGATATAGGCCTCCCTCAGCCCTTCCT

TTTTGTGCGGTTCCGTCTCCTACCCAGCTCAGCCTCTTCTCCCCCGCTCAGACAGGGGTC

CCCATCACATGCCGCTCTCTGAGCGACCTCTCCATAGGCCTTCGCTGGCCTCAGAGCCCC

TCCCTGCGTGTCCTTCCCCTGGCGGACTGCCTTCTCCCACATCGTCGAATTCCTTTCCCCG

GGTTCTACGGCCCCGCCGCTCCTCCCACCATCTCTCTTTTCGGGTGTAGCGCCCCCTCCC

CCTCGGCGTACACCCTTCCCAGCTCGCGTCCTCTCCCGAAGCCCCTCTG

HSP90AA1 GS-puromycin-V5 tag knock-in HDR template sequence

Left Homology Arm-Insertion Sequence-Right Homology Arm

(Underlined are the inserted GS-puromycin-V5 tag protein sequence)

GGCTGGACAGCAAACATGGAGAGAATCATGAAAGCTCAAGCCCTAAGAGACAACTCAACA

ATGGGTTACATGGCAGCAAAGAAACACCTGGAGATAAACCCTGACCATTCCATTATTGAGA

CCTTAAGGCAAAAGGCAGAGGCTGATAAGAACGACAAGTCTGTGAAGGATCTGGTCATCTT

GCTTTATGAAACTGCGCTCCTGTCTTCTGGCTTCAGTCTGGAAGATCCCCAGACACATGCT

AACAGGATCTACAGGATGATCAAACTTGGTCTGGGTAAGCCTTATACTATGTAATGTTAAAA

AGAAAATAAACACACGTGACATTGAAGAAAATGGTGAACTTTCAGTTATCCAAACTTGGAGC
ACCTTGTCCTGCTTGCTGCTTGGAGGTATTAAAGTATGTTTTTTTAGGGATAAGTAAGGTC
TTACAAGAGCAAAGAAATGAAATTGAGACTCATATGTCCTGTAATACTGTCTTGAAAGCAGA
TAGAAACCAAGAGTATTACCCTAATAGCTGGCTTTAAGAAATCTTTGTAATATGAGGATTTTA
TTTTGGAAACAGGTATTGATGAAGATGACCCTACTGCTGATGATACCAGTGCTGCTGTAAC
TGAAGAAATGCCACCCCTTGAAGGAGATGACGACACATCACGCATGGAAGAAGTAGACGG
AGGAGGTGGAAGCGGAGGAGGAGGAAGCATGACCGAGTACAAGCCCACGGTGCGCCTC
GCCACCCGCGACGACGTCCCCAGGGCCGTACGCACCCTCGCCGCCGCGTTCGCCGACTA
CCCCGCCACGCGCCACACCGTCGATCCGGACCGCCACATCGAGCGGGTCACCGAGCTGC
AAGAACTCTTCCTCACGCGCGTCGGGCTCGACATCGGCAAGGTGTGGGTCGCGGACGAC
GGCGCCGCGGTGGCGGTCTGGACCACGCCGGAGAGCGTCGAAGCGGGGGCGGTGTTCG
CCGAGATCGGCCCGCGCATGGCCGAGTTGAGCGGTTCCCGGCTGGCCGCGCAGCAACA
GATGGAAGGCCTCCTGGCGCCGCACCGGCCCAAGGAGCCCGCGTGGTTCCTGGCCACC
GTCGGCGTCTCGCCCGACCACCAGGGCAAGGGTCTGGGCAGCGCCGTCGTGCTCCCCG
GAGTGGAGGCGGCCGAGCGCGCCGGGGTGCCCGCCTTCCTGGAGACCTCCGCGCCCCG
CAACCTCCCCTTCTACGAGCGGCTCGGCTTCACCGTCACCGCCGACGTCGAGGTGCCCG
AAGGACCGCGCACCTGGTGCATGACCCGCAAGCCCGGTGCCGCCGGCTCCGGTACCGGT
AAGCCTATCCCTAACCCTCTCCTCGGTCTCGATTCTACGTAATCTGTGGCTGAGGGATGAC
TTACCTGTTCAGTACTCTACAATTCCTCTGATAATATATTTTCAAGGATGTTTTTCTTTATTTT
TGTTAATATTAAAAAGTCTGTATGGCATGACAACTACTTTAAGGGGAAGATAAGATTTCTGT
CTACTAAGTGATGCTGTGATACCTTAGGCACTAAAGCAGAGCTAGTAATGCTTTTTGAGTTT
CATGTTGGTTTATTTTCACAGATTGGGGTAACGTGCACTGTAAGACGTATGTAACATGATGT
TAACTTTGTGGTCTAAAGTGTTTAGCTGTCAAGCCGGATGCCTAAGTAGACCAAATCTTGTT
ATTGAAGTGTTCTGAGCTGTATCTTGATGTTTAGAAAAGTATTCGTTACATCTTGTAGGATCT
ACTTTTTGAACTTTTCATTCCCTGTAGTTGACAATTCTGCATGTACTAGTCCTCTAGAAATAG
GTTAAACTGAAGCAACTTGATGGAAGGATCTCTCCACAGGGCTTGTTTTCCAAAGAAAAGT

ATTGTTTGGAGGAGCAAAGTTAAAAGCCTACCTAAGCATATCGTAAAGCTGTTCAAAAATAA

CTCAGACCCAGTCTTGTGGATGGAAATGTAGTGCTCGAGTCACATTCTGCTTAAAGTTGTA

ACAAATACAGATGAGTTAAAAGATATTGTGTGACAGTGTCTTATTTAGGGGGAAAGGGGAG

TATCTGGATGACAGTTAGTGCCAAAATGTAAAACATGAGGCGCTAGCAGGAGAT

HSP90AA1 GS-BSD-V5 tag knock-in HDR template sequence

Left Homology Arm-Insertion Sequence-Right Homology Arm

(Underlined are the inserted GS-BSD-V5 tag protein sequence)

GGCTGGACAGCAAACATGGAGAGAATCATGAAAGCTCAAGCCCTAAGAGACAACTCAACA

ATGGGTTACATGGCAGCAAAGAAACACCTGGAGATAAACCCTGACCATTCCATTATTGAGA

CCTTAAGGCAAAAGGCAGAGGCTGATAAGAACGACAAGTCTGTGAAGGATCTGGTCATCTT

GCTTTATGAAACTGCGCTCCTGTCTTCTGGCTTCAGTCTGGAAGATCCCCAGACACATGCT

AACAGGATCTACAGGATGATCAAACTTGGTCTGGGTAAGCCTTATACTATGTAATGTTAAAA

AGAAAATAAACACACGTGACATTGAAGAAAATGGTGAACTTTCAGTTATCCAAACTTGGAGC

ACCTTGTCCTGCTTGCTGCTTGGAGGTATTAAAGTATGTTTTTTTTAGGGATAAGTAAGGTC

TTACAAGAGCAAAGAAATGAAATTGAGACTCATATGTCCTGTAATACTGTCTTGAAAGCAGA

TAGAAACCAAGAGTATTACCCTAATAGCTGGCTTTAAGAAATCTTTGTAATATGAGGATTTTA

TTTTGGAAACAGGTATTGATGAAGATGACCCTACTGCTGATGATACCAGTGCTGCTGTAAC

TGAAGAAATGCCACCCCTTGAAGGAGATGACGACACATCACGCATGGAAGAAGTAGACGG

AGGAGGTGGAAGCGGAGGAGGAGGAAGCATGGCCAAGCCTTTGTCTCAAGAAGAATCCA

CCCTCATTGAAAGAGCAACGGCTACAATCAACAGCATCCCCATCTCTGAAGACTACAGCGT

CGCCAGCGCAGCTCTCTCTAGCGACGGCCGCATCTTCACTGGTGTCAATGTATATCATTTT

ACTGGGGGACCTTGTGCAGAACTCGTGGTGCTGGGCACTGCTGCTGCTGCGGCAGCTGG

CAACCTGACTTGTATCGTCGCGATCGGAAATGAGAACAGGGGCATCTTGAGCCCCTGCGG
ACGGTGCCGACAGGTGCTTCTCGATCTGCATCCTGGGATCAAAGCCATAGTGAAGGACAG
TGATGGACAGCCGACGGCAGTTGGGATTCGTGAATTGCTGCCCTCTGGTTATGTGTGGGA
GGGCCTGCAGCTGCAGGCCGGCTCCGGTACCGGTAAGCCTATCCCTAACCCTCTCCTCG
GTCTCGATTCTACGTAATCTGTGGCTGAGGGATGACTTACCTGTTCAGTACTCTACAATTCC
TCTGATAATATATTTTCAAGGATGTTTTTCTTTATTTTTGTTAATATTAAAAAGTCTGTATGGC
ATGACAACTACTTTAAGGGGAAGATAAGATTTCTGTCTACTAAGTGATGCTGTGATACCTTA
GGCACTAAAGCAGAGCTAGTAATGCTTTTTGAGTTTCATGTTGGTTTATTTTCACAGATTGG
GGTAACGTGCACTGTAAGACGTATGTAACATGATGTTAACTTTGTGGTCTAAAGTGTTTAGC
TGTCAAGCCGGATGCCTAAGTAGACCAAATCTTGTTATTGAAGTGTTCTGAGCTGTATCTTG
ATGTTTAGAAAAGTATTCGTTACATCTTGTAGGATCTACTTTTTGAACTTTTCATTCCCTGTA
GTTGACAATTCTGCATGTACTAGTCCTCTAGAAATAGGTTAAACTGAAGCAACTTGATGGAA
GGATCTCTCCACAGGGCTTGTTTTCCAAAGAAAAGTATTGTTTGGAGGAGCAAAGTTAAAA
GCCTACCTAAGCATATCGTAAAGCTGTTCAAAAATAACTCAGACCCAGTCTTGTGGATGGA
AATGTAGTGCTCGAGTCACATTCTGCTTAAAGTTGTAACAAATACAGATGAGTTAAAAGATA
TTGTGTGACAGTGTCTTATTTAGGGGGAAAGGGGAGTATCTGGATGACAGTTAGTGCCAAA
ATGTAAAACATGAGGCGCTAGCAGGAGAT

ACTB GS-puromycin-V5 tag knock-in HDR template sequence

Left Homology Arm-Insertion Sequence-Right Homology Arm

(Underlined are the inserted GS-puromycin-V5 tag protein sequence)

CGGCTCTGCCTGACATGAGGGTTACCCCTCGGGGCTGTGCTGTGGAAGCTAAGTCCTGCC
CTCATTTCCCTCTCAGGCATGGAGTCCTGTGGCATCCACGAAACTACCTTCAACTCCATCA

TGAAGTGTGACGTGGACATCCGCAAAGACCTGTACGCCAACACAGTGCTGTCTGGCGGCA
CCACCATGTACCCTGGCATTGCCGACAGGATGCAGAAGGAGATCACTGCCCTGGCACCCA
GCACAATGAAGATCAAGGTGGGTGTCTTTCCTGCCTGAGCTGACCTGGGCAGGTCGGCTG
TGGGGTCCTGTGGTGTGTGGGGAGCTGTCACATCCAGGGTCCTCACTGCCTGTCCCCTTC
CCTCCTCAGATCATTGCTCCTCCTGAGCGCAAGTACTCCGTGTGGATCGGCGGCTCCATC
CTGGCCTCGCTGTCCACCTTCCAGCAGATGTGGATCAGCAAGCAGGAGTATGACGAGTCC
GGCCCCTCCATCGTCCACCGCAAGTGTTTCGGAGGAGGTGGAAGCGGAGGAGGAGGAAG
CATGACCGAGTACAAGCCCACGGTGCGCCTCGCCACCCGCGACGACGTCCCCAGGGCCG
TACGCACCCTCGCCGCCGCGTTCGCCGACTACCCCGCCACGCGCCACACCGTCGATCCG
GACCGCCACATCGAGCGGGTCACCGAGCTGCAAGAACTCTTCCTCACGCGCGTCGGGCT
CGACATCGGCAAGGTGTGGGTCGCGGACGACGGCGCCGCGGTGGCGGTCTGGACCACG
CCGGAGAGCGTCGAAGCGGGGGCGGTGTTCGCCGAGATCGGCCCGCGCATGGCCGAGT
TGAGCGGTTCCCGGCTGGCCGCGCAGCAACAGATGGAAGGCCTCCTGGCGCCGCACCG
GCCCAAGGAGCCCGCGTGGTTCCTGGCCACCGTCGGCGTCTCGCCCGACCACCAGGGCA
AGGGTCTGGGCAGCGCCGTCGTGCTCCCCGGAGTGGAGGCGGCCGAGCGCGCCGGGGT
GCCCGCCTTCCTGGAGACCTCCGCGCCCCGCAACCTCCCCTTCTACGAGCGGCTCGGCT
TCACCGTCACCGCCGACGTCGAGGTGCCCGAAGGACCGCGCACCTGGTGCATGACCCGC
AAGCCCGGTGCCGCCGGCTCCGGTACCGGTAAGCCTATCCCTAACCCTCTCCTCGGTCTC
GATTCTACGTAATAGGCGGACTATGACTTAGTTGCGTTACACCCTTTCTTGACAAAACCTAA
CTTGCGCAGAAAACAAGATGAGATTGGCATGGCTTTATTTGTTTTTTTTGTTTTGTTTTGGTT
TTTTTTTTTTTTTTGGCTTGACTCAGGATTTAAAAACTGGAACGGTGAAGGTGACAGCAGTC
GGTTGGAGCGAGCATCCCCCAAAGTTCACAATGTGGCCGAGGACTTTGATTGCACATTGTT
GTTTTTTTAATAGTCATTCCAAATATGAGATGCGTTGTTACAGGAAGTCCCTTGCCATCCTA
AAAGCCACCCCACTTCTCTCTAAGGAGAATGGCCCAGTCCTCTCCCAAGTCCACACAGGG
GAGGTGATAGCATTGCTTTCGTGTAAATTATGTAATGCAAAATTTTTTTAATCTTCGCCTTAA
TACTTTTTTATTTTGTTTTATTTTGAATGATGAGCCTTCGTGCCCCCCCTTCCCCCTTTTTTG

TCCCCCAACTTGAGATGTATGAAGGCTTTTGGTCTCCCTGGGAGTGGGTGGAGGCAGCCA
GGGCTTACCTGTACACTGACTTGAGACCAGTTGAATAAAAGTGCACACCTTAAAAATGAGG
CCAAGTGTGACTTTGTGGTGTGGCTGGGTTGGGGGCAGCAGAGGGTGAACCCTGCAGGA
GGGTGAACCCTGCAAAAGGGTGGGGCAGTGGGGGCCAACTTGTCCTTACCCAGAGTGCA
GGTGTGTGGAGATCCCTCCTGCCTTGACATTGAGCAGCCTTAGAGGGTGGGGGAGGCTCA
GGGGTCAGGTCTCTGTTC

Supplementary references

66. S. Nakade et al., Microhomology-mediated end-joining-dependent integration of donor DNA in cells and animals using TALENs and CRISPR/Cas9. Nat Commun 5, 5560 (2014).

67. A. Paix et al., Precision genome editing using synthesis-dependent repair of Cas9-induced DNA breaks. Proc Natl Acad Sci U S A 114, E10745-E10754 (2017).

68. O. Kanca et al., An efficient CRISPR-based strategy to insert small and large fragments of DNA using short homology arms. Elife 8, (2019).

69. K. J. Tatiossian et al., Rational Selection of CRISPR-Cas9 Guide RNAs for Homology-Directed Genome Editing. Mol Ther 29, 1057-1069 (2021).
