# Supplementary material for: dCas9-based gene editing for cleavage-free genomic knock-in of long sequences
Source: Nat Cell Biol. 2022 Feb 10;24(2):268–78. doi: 10.1038/s41556-021-00836-1 (PMC8843813; doi:10.1038/s41556-021-00836-1)
Supplement: Supplementary file 2 — Reporting Summary [file 41556_2021_836_MOESM2_ESM.pdf]

nature research

Corresponding author(s): Le Cong

Last updated by author(s): Jun 17, 2021

Reporting Summary

Nature Research wishes to improve the reproducibility of the work that we publish. This form provides structure for consistency and transparency in reporting. For further information on Nature Research policies, see our Editorial Policies and the Editorial Policy Checklist.

Statistics

For all statistical analyses, confirm that the following items are present in the figure legend, table legend, main text, or Methods section.

n/a	Confirmed	
☐	☒	The exact sample size (n) for each experimental group/condition, given as a discrete number and unit of measurement
☐	☒	A statement on whether measurements were taken from distinct samples or whether the same sample was measured repeatedly
☐	☒	The statistical test(s) used AND whether they are one- or two-sided<br>*Only common tests should be described solely by name; describe more complex techniques in the Methods section.*
☒	☐	A description of all covariates tested
☐	☒	A description of any assumptions or corrections, such as tests of normality and adjustment for multiple comparisons
☐	☒	A full description of the statistical parameters including central tendency (e.g. means) or other basic estimates (e.g. regression coefficient) AND variation (e.g. standard deviation) or associated estimates of uncertainty (e.g. confidence intervals)
☐	☒	For null hypothesis testing, the test statistic (e.g. F, t, r) with confidence intervals, effect sizes, degrees of freedom and P value noted<br>*Give P values as exact values whenever suitable.*
☒	☐	For Bayesian analysis, information on the choice of priors and Markov chain Monte Carlo settings
☒	☐	For hierarchical and complex designs, identification of the appropriate level for tests and full reporting of outcomes
☒	☐	Estimates of effect sizes (e.g. Cohen's d, Pearson's r), indicating how they were calculated

Our web collection on statistics for biologists contains articles on many of the points above.

Software and code

Policy information about availability of computer code

Data collection	For the FACS readout, we used Cytoflex FACS analyzer for all data collection.<br>We used the Keyence Microscope to take Crystal Violet images acquired with a BZ-X700 system.
Data analysis	CytExpert v2.3 and Flowjo v10.7.2. was used for all the FACS results analysis.<br>ImageJ/Fiji v1.0 was used for the Crystal Violet and Western Blot data analysis.<br>CRISPResso2 was used for all next-generation sequencing analysis to measure gene-editing efficiencies, using version 2.0.29.<br><br>Customized scripts for data analysis are deposited and available at GitHub under Cong Lab repository (https://github.com/cong-lab).

For manuscripts utilizing custom algorithms or software that are central to the research but not yet described in published literature, software must be made available to editors and reviewers. We strongly encourage code deposition in a community repository (e.g. GitHub). See the Nature Research guidelines for submitting code & software for further information.

Data

Policy information about availability of data

All manuscripts must include a data availability statement. This statement should provide the following information, where applicable:

- Accession codes, unique identifiers, or web links for publicly available datasets
- A list of figures that have associated raw data
- A description of any restrictions on data availability

Data for next-generation sequencing experiments are accessible via the NCBI Sequence Read Archive database with accession code PRJNA683925, or are available from the corresponding author upon reasonable request.

Field-specific reporting

Please select the one below that is the best fit for your research. If you are not sure, read the appropriate sections before making your selection.

☒ Life sciences ☐ Behavioural & social sciences ☐ Ecological, evolutionary & environmental sciences

For a reference copy of the document with all sections, see nature.com/documents/nr-reporting-summary-flat.pdf

Life sciences study design

All studies must disclose on these points even when the disclosure is negative.

Sample size

In this work, all gene-editing experiments on endogenous genome loci were performed with at least two biologically independent replicate experiments with negative controls, and for measuring gene-editing efficiencies at least three independent replicates were used. The numbers of replicates was listed in the text and figure legends when applicable. For all cell survival experiments, at least four independent replicates were used based on the current standard of the field.

The group sizes in all imaging and flow cytometry experiments were selected based on the prior knowledge of variation and experimental noises to detect specific effects, with at least three independent replicates and detailed in the material and methods section and in figure legends when applicable.

Data exclusions

No data were excluded.

Replication

The numbers of experimental replicates are listed and detailed in material and methods section, and in figure legends when applicable. All attempts at replication were successful.

Randomization

For all experiments, cells were treated with replicates and vehicle-only control were included to control for possible background. For all flow cytometry analysis, cells were transfected with replicates, the samples were randomly allocated and analyzed according to the fluorescence marker panel design with proper compensation. To control for possible background and noises, in all flow cytometry experiments, negative control where cells were mock-transfected, and blank group without any treatment were included.

Blinding

For all experiments in this project, the investigator were blinded to the group allocation during data collection and data analysis.

Reporting for specific materials, systems and methods

We require information from authors about some types of materials, experimental systems and methods used in many studies. Here, indicate whether each material, system or method listed is relevant to your study. If you are not sure if a list item applies to your research, read the appropriate section before selecting a response.

Materials & experimental systems

n/a	Involved in the study	
☐	☒	Antibodies
☐	☒	Eukaryotic cell lines
☒	☐	Palaeontology and archaeology
☒	☐	Animals and other organisms
☒	☐	Human research participants
☒	☐	Clinical data
☒	☐	Dual use research of concern

Methods

n/a	Involved in the study	
☒	☐	ChIP-seq
☐	☒	Flow cytometry
☒	☐	MRI-based neuroimaging

Antibodies

Antibodies used

V5 tag monoclonal antibody (Thermo Scientific, R960-25) dilution of 1:2000 for all western blot experiments.

Validation

This Antibody (R960-25) is an "Advanced Verification" product by ThermoFisher, and was verified by Relative expression to ensure that the antibody binds to the antigen stated. This antibody has been cited over 796 times in literature and validated by over 479 publications for western blot (e.g. Pathria G, et al. Translational reprogramming marks adaptation to asparagine restriction in cancer. Nat Cell Biol. 2019. PMID: 31740775).

Specifically, the Antibody specificity was demonstrated by detection of different targets fused to V5 tag in transiently transfected lysates tested. Relative detection of V5 tag was observed across different proteins fused with V5 tag in V5-H3-His and Myc-p65-V5, using V5 Tag Monoclonal Antibody (Product # R960-25) in Western Blot (https://www.thermofisher.com/antibody/product/V5-Tag-Antibody-Monoclonal/R960-25). This product has been shown to detect V5 Tag at both N- and C- termini of a fusion protein.

Eukaryotic cell lines

Policy information about cell lines

Cell line source(s)	HEK 293T, Hela, HepG2 and U2OS cells were obtained from American Type Culture Collection (ATCC). Human embryonic stem cells hES-H9 were obtained from Stanford Stem Cell Core Facility.
Authentication	STR analysis was used for cell line authentication.
Mycoplasma contamination	All cells were tested negative for mycoplasma contamination.
Commonly misidentified lines (See ICLAC register)	No commonly misidentified cell lines were used.

Flow Cytometry

Plots

Confirm that:

☒ The axis labels state the marker and fluorochrome used (e.g. CD4-FITC).

☒ The axis scales are clearly visible. Include numbers along axes only for bottom left plot of group (a 'group' is an analysis of identical markers).

☒ All plots are contour plots with outliers or pseudocolor plots.

☒ A numerical value for number of cells or percentage (with statistics) is provided.

Methodology

Sample preparation	Cells were transfected using Lipofectamine 3000 (Life Technologies) following the manufacturer's instructions. 3 days after transfection, cells were washed once with PBS and dissociated with TrypLE Express Enzyme (Thermo Fisher Scientific). Cell suspension was then transferred to a 96-well U-bottom plate (Thermo Fisher Scientific) and centrifuged at 300g for 5 minutes. After removing the supernatant, pelleted cells were resuspended with 50 µl 4% FBS in PBS, and cells were analyzed within 30 minutes of preparation.
Instrument	All samples were analyzed on a CytoFLEX flow cytometer (Stanford Stem Cell FACS Core).
Software	Beckman Coulter CytoExpert, followed by secondary analysis using FlowJo.
Cell population abundance	All fluorescence proteins (EBFP, mKate or EGFP) used was either encoded in the plasmids used for transfection or present as genomic knock-in cassette after successful gene-editing. For all experiments, a minimum of 1E5 cells were collected for downstream analysis.
Gating strategy	After data collection, FCS files were imported into FlowJo softeware for gating and analysis. Starting cell populations were selected according to SSC-A, FSC-A, and then single-cell were gated using FSC-A, FSC-W gate. Then positive cell populations were determined according to the negative control groups (non-targeting controls in related figures). As a purity check, mock-transfected controls were also included in the analysis.

☒ Tick this box to confirm that a figure exemplifying the gating strategy is provided in the Supplementary Information.
