# Supplementary figures and images for: dCas9-based gene editing for cleavage-free genomic knock-in of long sequences
Source: Nat Cell Biol. 2022 Feb 10;24(2):268–78. doi: 10.1038/s41556-021-00836-1 (PMC8843813; doi:10.1038/s41556-021-00836-1)

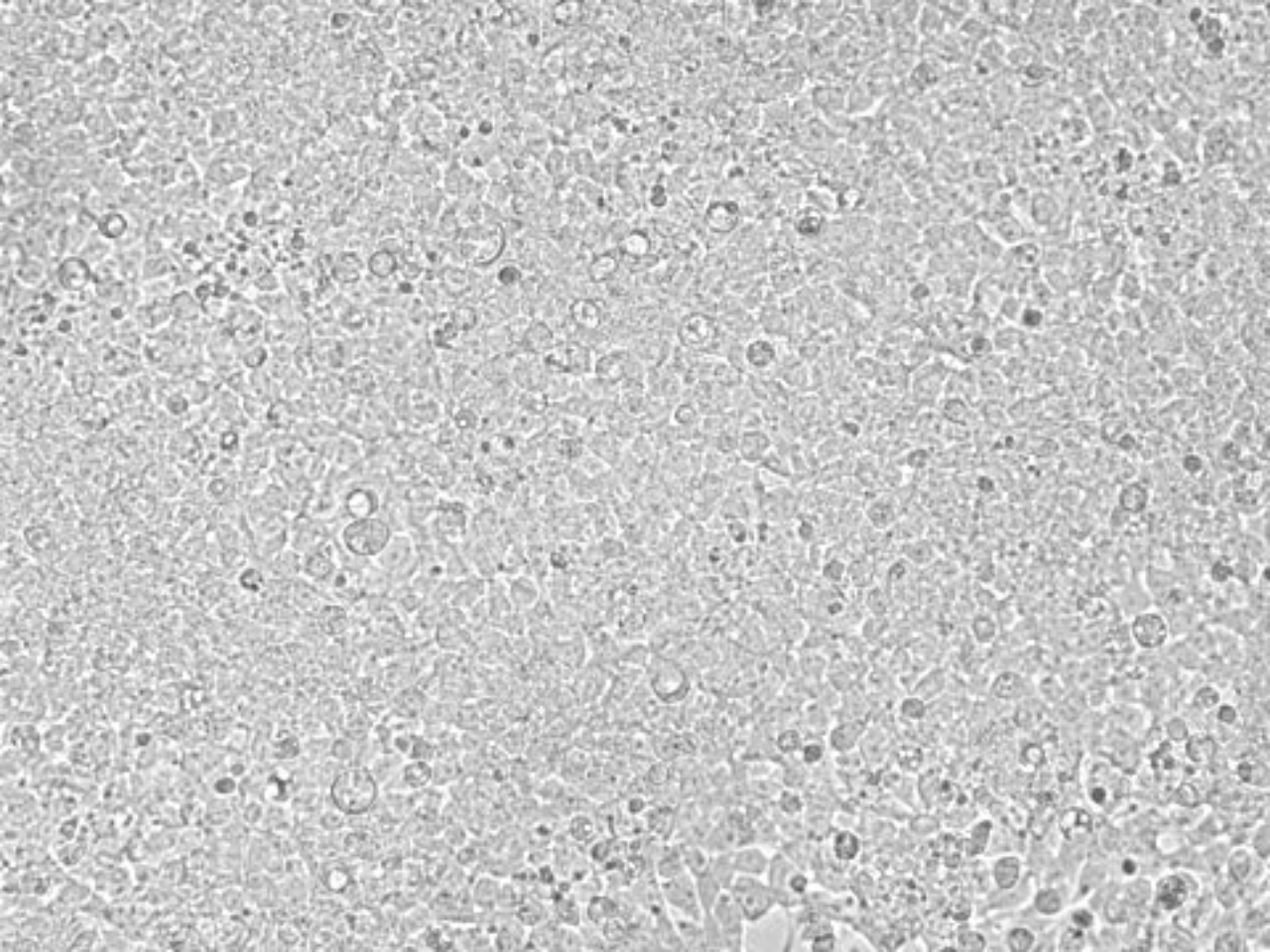



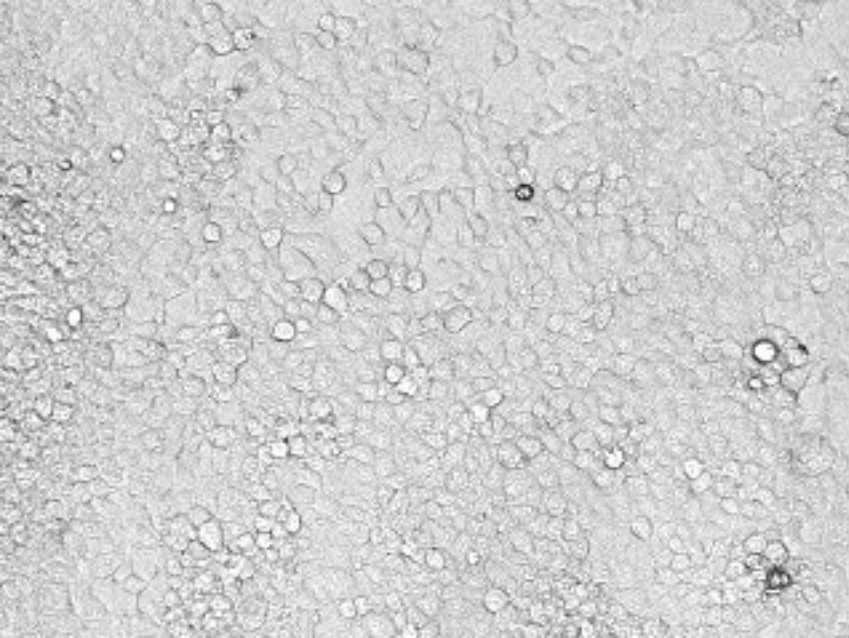

Supplement: Source Data Fig. 1 — Unprocessed images. [file 41556_2021_836_MOESM5_ESM.pdf]

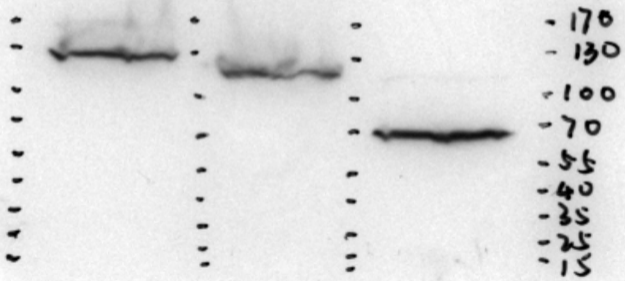



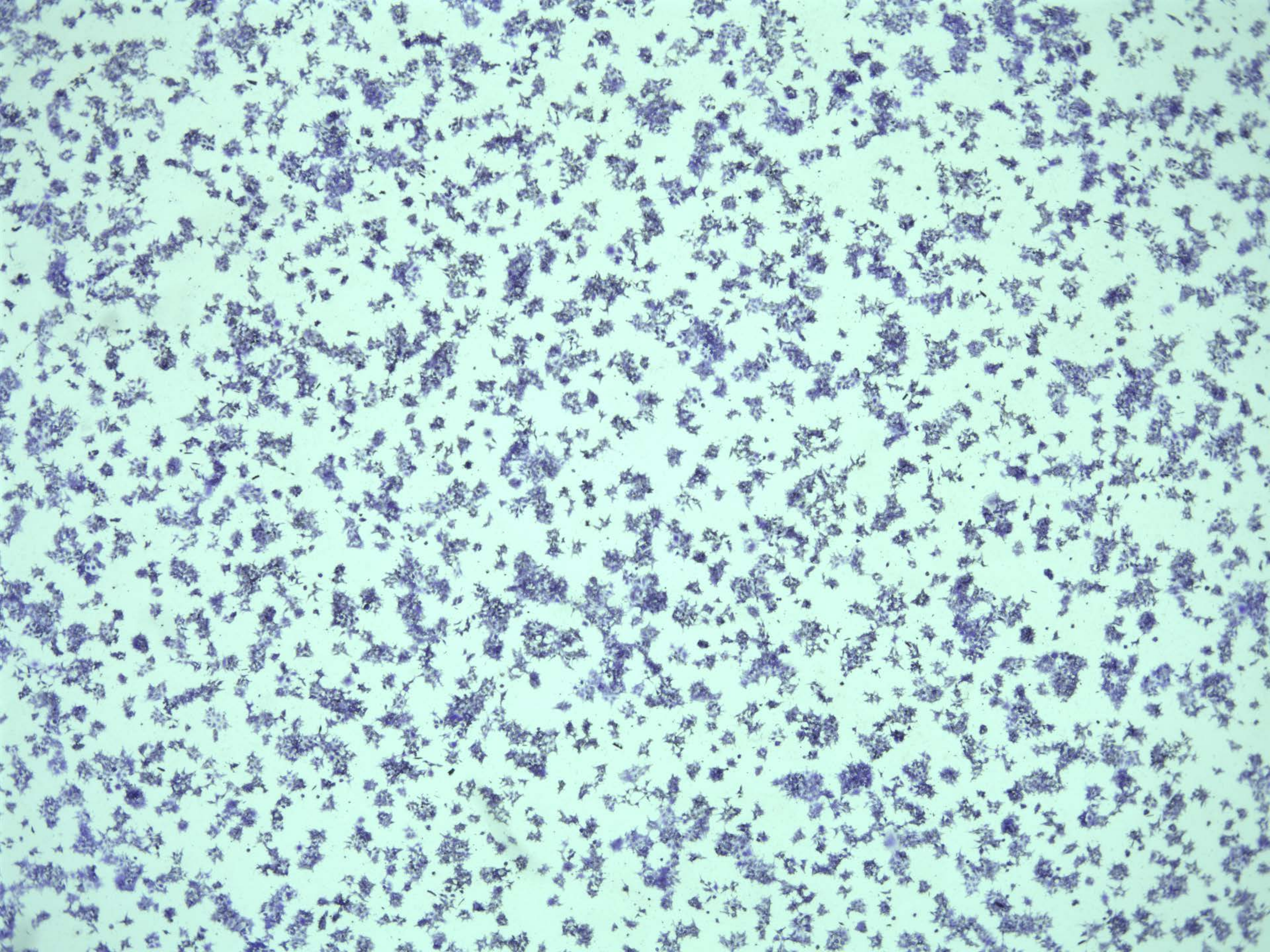



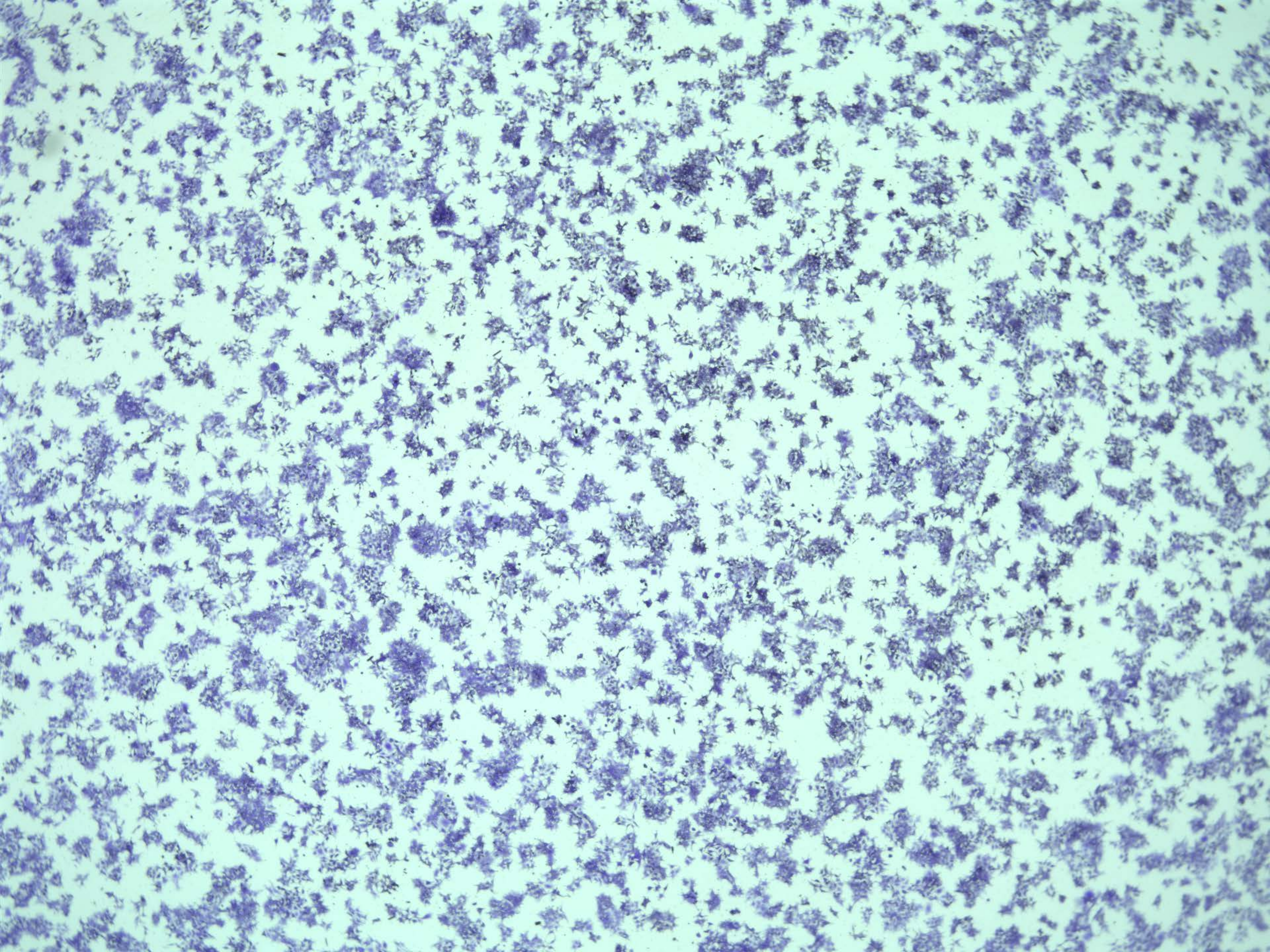

Supplement: Source Data Fig. 5 — Unprocessed images and unprocessed western blots. [file 41556_2021_836_MOESM10_ESM.pdf]

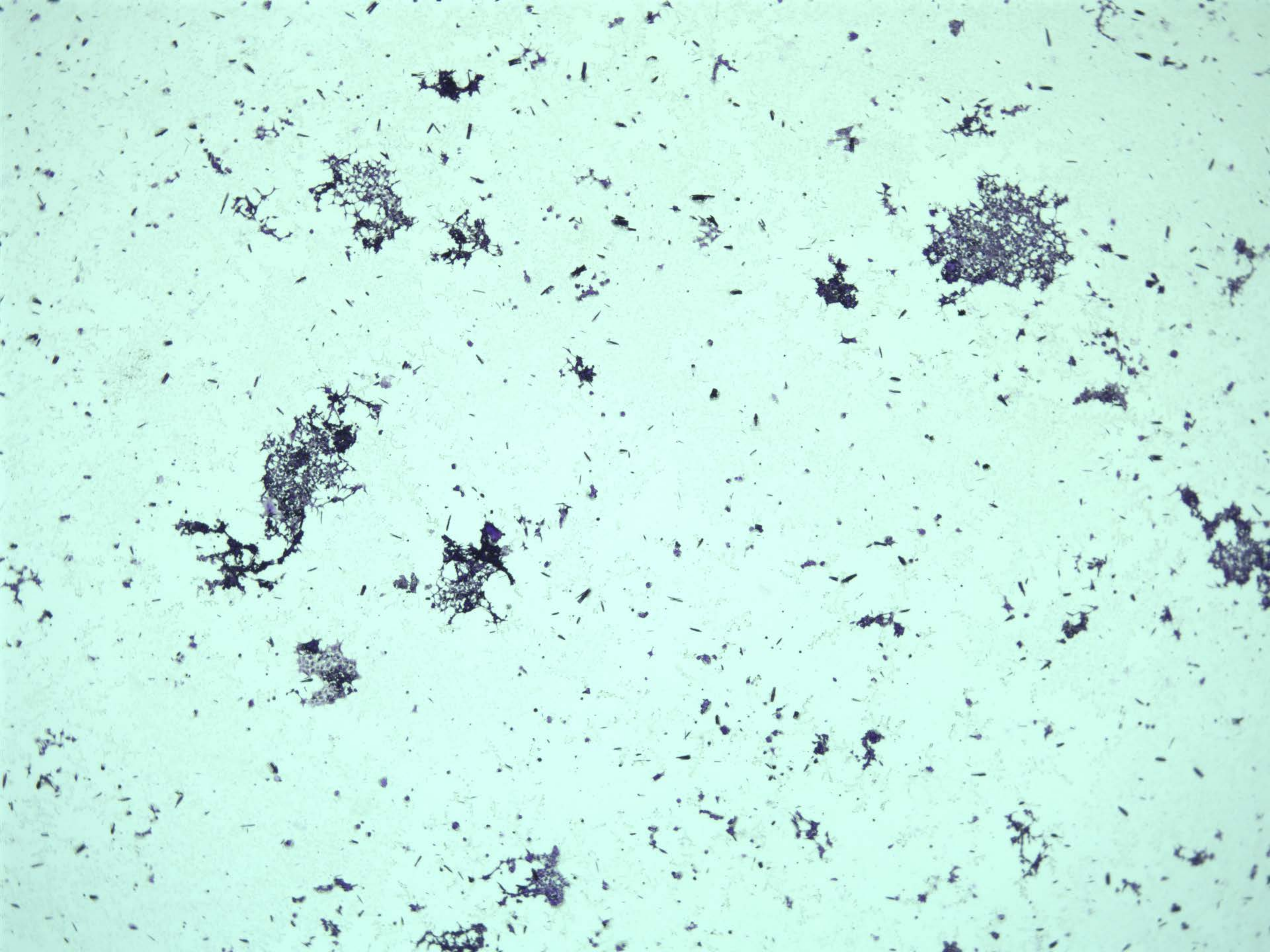

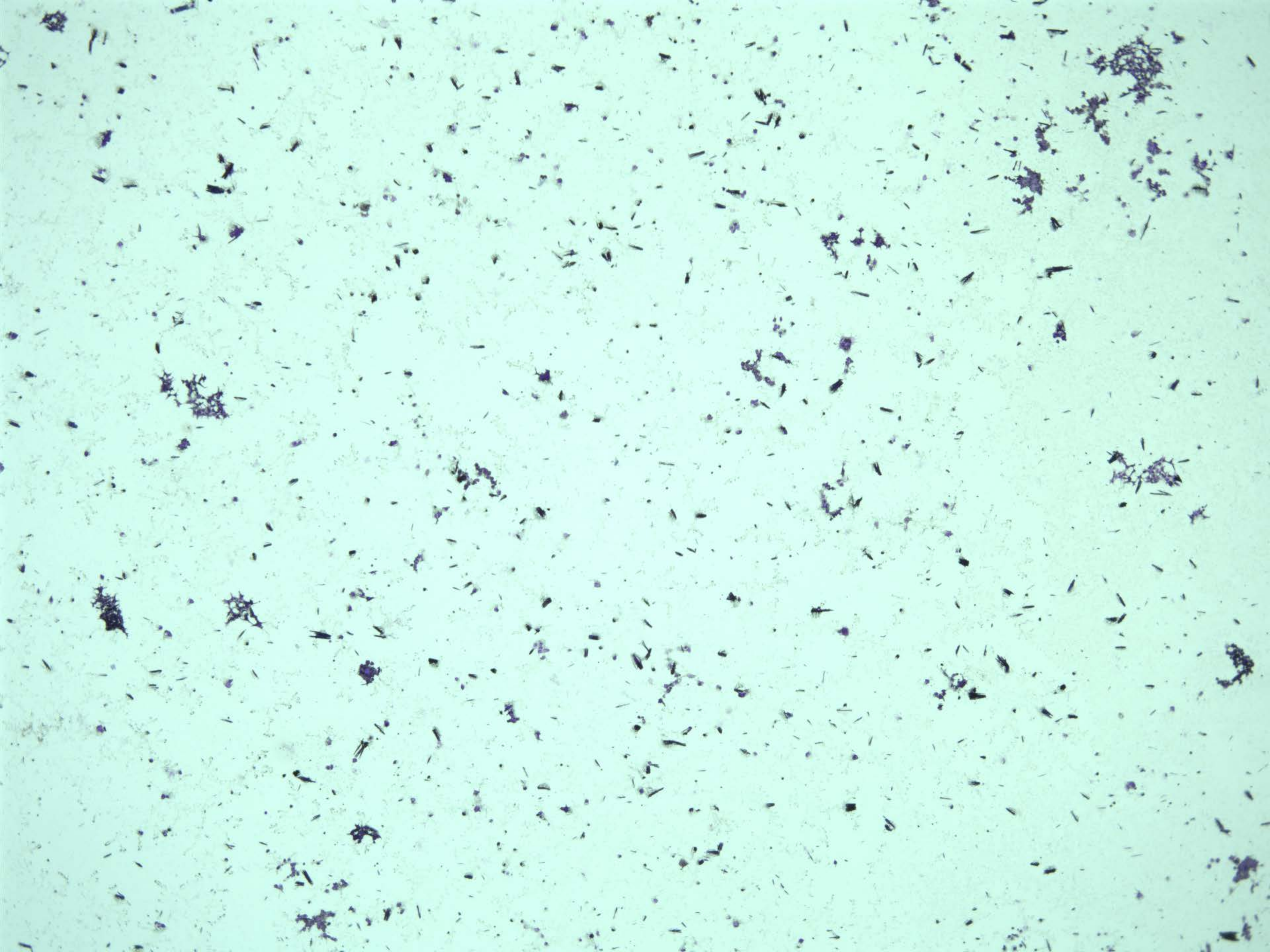

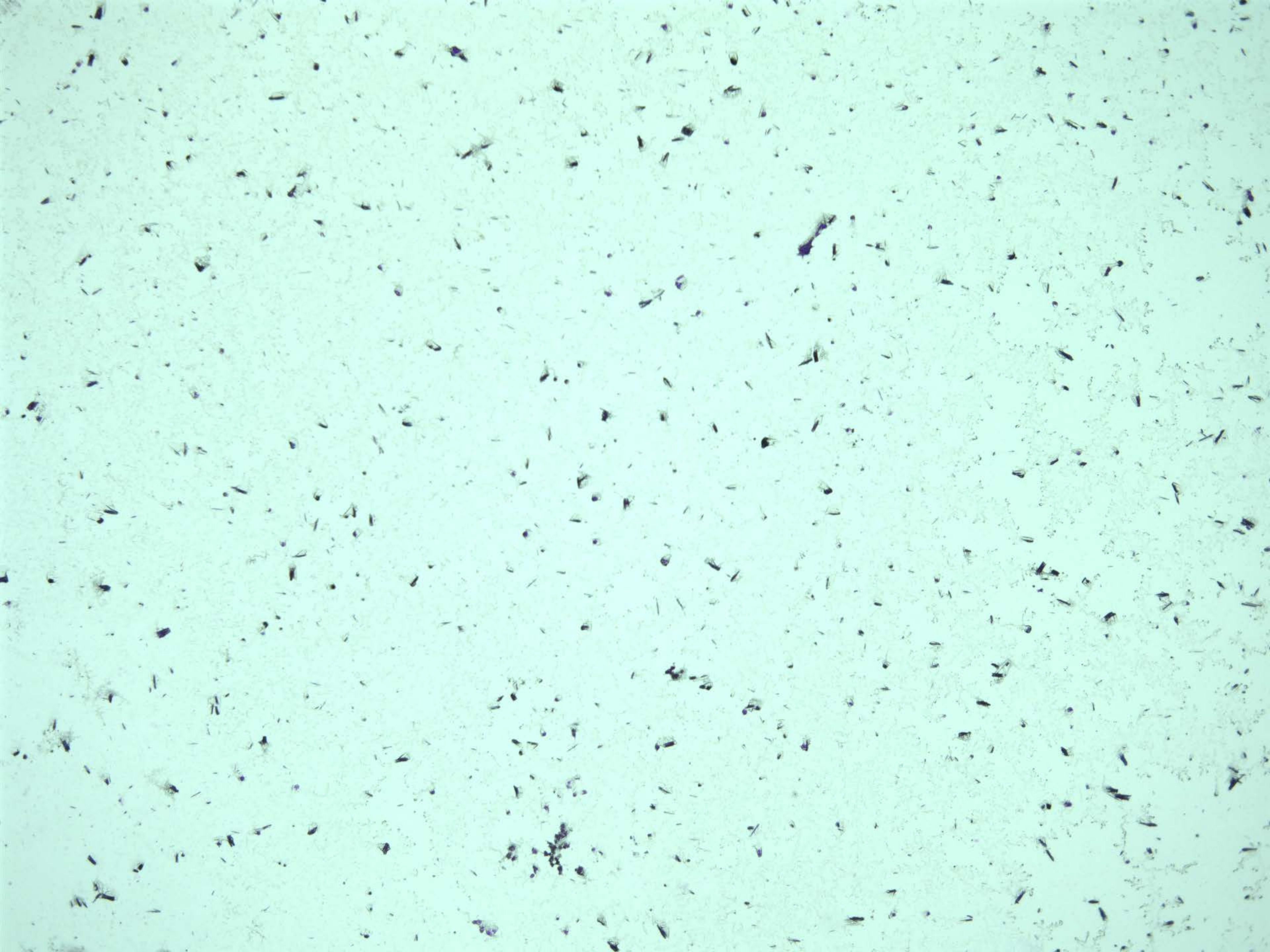

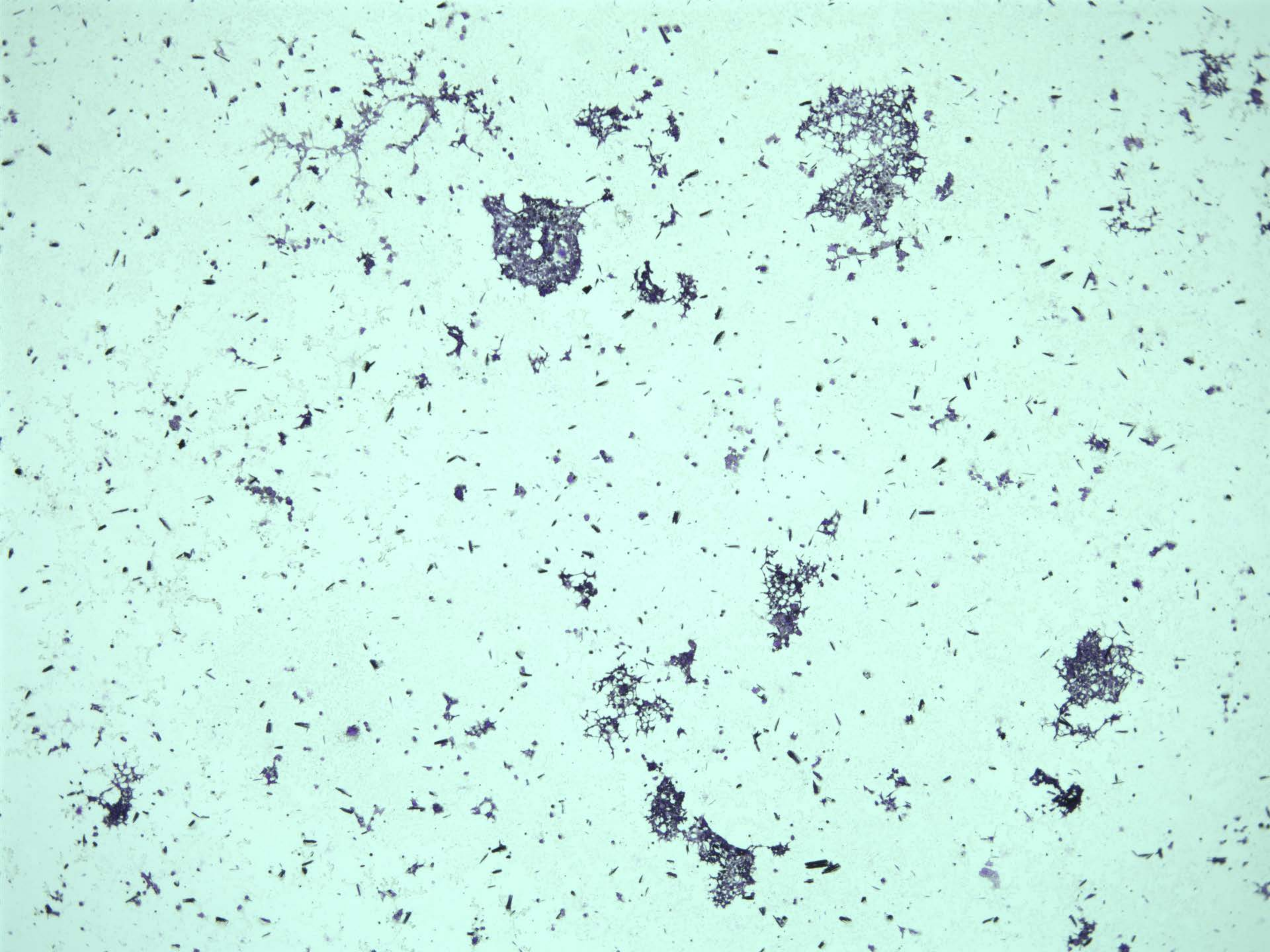

Supplement: Source Data Extended Data Fig. 9 — Unprocessed images. [file 41556_2021_836_MOESM19_ESM.pdf]
